# The small and large intestine contain related mesenchymal subsets that derive from embryonic *Gli1*^*+*^ precursors

**DOI:** 10.1038/s41467-023-37952-5

**Published:** 2023-04-21

**Authors:** Simone Isling Pærregaard, Line Wulff, Sophie Schussek, Kristoffer Niss, Urs Mörbe, Johan Jendholm, Kerstin Wendland, Anna T. Andrusaite, Kevin F. Brulois, Robert J. B. Nibbs, Katarzyna Sitnik, Allan McI Mowat, Eugene C. Butcher, Søren Brunak, William W. Agace

**Affiliations:** 1 Department of Health Technology, Technical University of Denmark, Kemitorvet, 2800 Kgs Lyngby, Denmark; 2 Novo Nordisk Foundation Center for Protein Research, Faculty of Health and Medical Sciences, University of Copenhagen, Copenhagen, 2200 Denmark; 3 Immunology Section, Lund University, Lund, 221 84 Sweden; 4 Institute of Infection, immunity and Inflammation, University of Glasgow, Glasgow, Scotland UK; 5 Laboratory of Immunology and Vascular Biology, Department of Pathology, School of Medicine, Stanford University, Stanford, CA USA; 6 The Center for Molecular Biology and Medicine, Veterans Affairs Palo Alto Health Care System and the Palo Alto Veterans Institute for Research (PAVIR), Palo Alto, CA USA

**Keywords:** Differentiation, Mesenchymal stem cells, RNA sequencing, Stem-cell differentiation

## Abstract

The intestinal lamina propria contains a diverse network of fibroblasts that provide key support functions to cells within their local environment. Despite this, our understanding of the diversity, location and ontogeny of fibroblasts within and along the length of the intestine remains incomplete. Here we show that the small and large intestinal lamina propria contain similar fibroblast subsets that locate in specific anatomical niches. Nevertheless, we find that the transcriptional profile of similar fibroblast subsets differs markedly between the small intestine and colon suggesting region specific functions. We perform in vivo transplantation and lineage-tracing experiments to demonstrate that adult intestinal fibroblast subsets, smooth muscle cells and pericytes derive from *Gli1*-expressing precursors present in embryonic day 12.5 intestine. Trajectory analysis of single cell RNA-seq datasets of E12.5 and adult mesenchymal cells suggest that adult smooth muscle cells and fibroblasts derive from distinct embryonic intermediates and that adult fibroblast subsets develop in a linear trajectory from CD81^+^ fibroblasts. Finally, we provide evidence that colonic subepithelial PDGFRα^hi^ fibroblasts comprise several functionally distinct populations that originate from an *Fgfr2*-expressing fibroblast intermediate. Our results provide insights into intestinal stromal cell diversity, location, function, and ontogeny, with implications for intestinal development and homeostasis.

## Abstract (web summary)

Stromal cells are essential for intestinal homeostasis. Here the authors describe the phenotype, transcriptional profile and location of stromal cell subsets in the adult murine small intestine and colon lamina propria and demonstrate that these cells derive from *Gli1+* precursors present in embryonic day 12.5 intestine.

## Introduction

The small and large intestines form a continuous tube from the stomach to the anus but are functionally and anatomically distinct. The small intestine is the primary site of food digestion and nutrient absorption and is characterized by finger-like projections termed villi that protrude into the intestinal lumen and maximize the absorptive area of the epithelium. In contrast, the large intestine is primarily a site of water absorption and is a major niche for beneficial microbes; its surface consists of crypts linked by short regions of flat surface epithelium. The cellular composition of the intestinal mucosa also differs markedly between the small and large intestines^1,2^. For example, the small and large intestines contain different numbers and proportions of innate and adaptive immune cells as well as epithelial subpopulations^1–4^. These distinct segments are also exposed to different concentrations of microbial and food-derived metabolites that regulate the composition and function of local cells^1,2^. However, the cellular and signaling components that determine the differences in tissue structure and composition are not fully understood.

The intestinal lamina propria (LP) contains a large population of tissue resident mesenchymal stromal cells (MSC) that include fibroblasts (FB), pericytes (PC) and smooth muscle cells (SMCs) that play an essential role in intestinal homeostasis^5–12^. For example, intestinal FB are major producers of extracellular matrix proteins that help provide structure to the mucosa^13,14^. They also express factors essential for epithelial^5–8,10,15–18^ and endothelial homeostasis^11,19,20^, as well as immune cell localization and function^21–24^. Recent single-cell (sc)RNA-seq studies have demonstrated considerable heterogeneity within the intestinal LP MSC compartment and have led to the identification of several FB clusters with non-redundant functions in intestinal homeostasis^5,7–9,11,12,25,26^. A picture is also emerging whereby different intestinal FB subsets locate within distinct regions of the mucosa^3,6,7,10,11,19,20^, providing specialized support to cells in their local environment^5–11,15,16,27,28^. Of particular interest has been determining the nature of the MSC that support the epithelium, with it being proposed that different MSC subsets may have distinct effects on stem cells and more differentiated epithelial cells depending on the location along the crypt-villus axis^7,9,10,27^. However, the extent to which the distinct environments of the small and large intestine influence the transcriptional profile and specialized functions of MSC subsets remains to be determined.

The composition of human intestinal MSC populations changes markedly as the tissue develops in the embryo^20,29^. Lineage-tracing experiments in mice have suggested that the mesothelium, a simple squamous epithelial layer that lines the serosal surface of the intestine^30^, undergoes epithelial to mesenchymal transition (EMT) in the developing embryo and can give rise to SMC and various FB in the intestinal serosa and muscle layers^31,32^. In contrast, the origin of the diverse MSC populations in the adult intestinal LP, whether these subsets derive from cells of common or distinct embryonic origin, as well as the developmental relationship between adult LP MSC subsets remains unclear.

Here we demonstrate that LP MSC subset composition was similar in the small and large intestine and that each subset occupies distinct anatomical niches. Nevertheless, the transcriptional profile of the major LP FB subsets differed markedly between the small and large intestine, suggesting regional specific functions in intestinal homeostasis. Grafting and lineage-tracing experiments demonstrated that all MSC subsets in adult small intestinal and colonic LP derive from *Gli1*-expressing precursors present in embryonic day (E)12.5 intestine. Computational analysis suggested that adult SMC and FB arise from distinct embryonic intermediates and defined a linear developmental trajectory for all adult FB subsets that originated from CD81^+^ FB.

## Results

### The small intestine and colon LP contain diverse, but transcriptionally related MSC subsets

To gain a broad understanding of MSC subset diversity in the intestinal LP, we performed scRNA-seq on MSC isolated from the small intestine and colon LP of 8–10-week-old mice. Briefly, after removal of Peyer’s patches, muscularis externa and epithelium, intestinal MSCs were enriched from digested intestinal LP cell suspensions by fluorescently activated cell sorting of live, single, lineage^−^ (CD45^+^, Ter119^+^), non-epithelial (EpCAM^+^), non-endothelial (CD31^+^), non-lymphoid tissue-associated MSCs (BP3^+^)^33^ and non-glial cells (L1CAM^+^), followed by gating on cells expressing the pan MSC marker Itgβ1 (Supplementary Fig. 1a). After bioinformatic removal of contaminating c-kit^+^ interstitial cells of Cajal (ICC), CD31^+^ endothelial cells, plasma cells and CD45^+^ immune cells, we were able to generate sequencing data of 16.964 small intestinal and 14.164 colonic MSC.

**Fig. 1:**
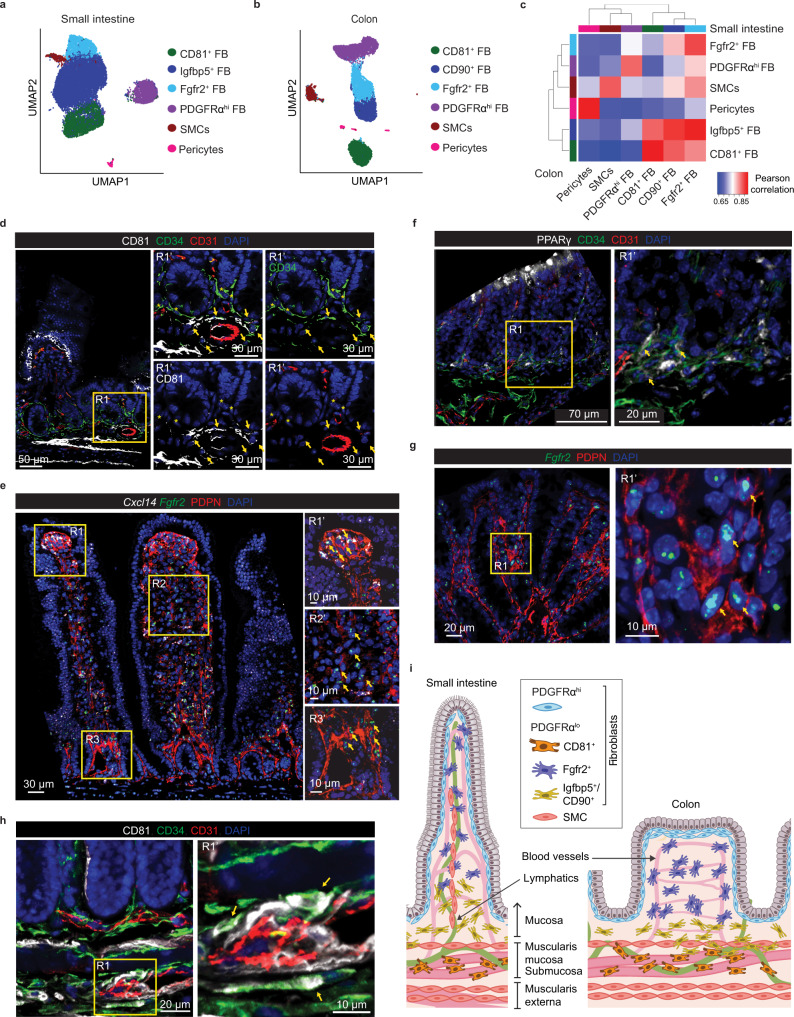
Intestinal MSC subsets are broadly conserved across intestinal segments. **a**, **b** Uniform Manifold Approximation and Projection (UMAP) colored by unsupervised Louvain clustering of murine small intestinal (**a**) and colonic (**b**) MSC. Results are from 2 independent experiments/organ with 3 pooled mice/experiment. **c** Pearson correlations between averaged cluster expressions of Louvain clusters from small intestinal and colonic MSC based on 1301 overlapping variable genes. Unsupervised hierarchical clustering indicates similarity of subsets within each tissue. **d**–**h** Immunohistochemical staining (**d**, **f**, **h**) or RNA-scope analysis (**e**, **g**) of mouse ileum (**d**, **e**) or colon (**f**–**h**). R, region; R′ represents magnifications of R quadrants (yellow squares). **d** Arrows indicate location of CD81^+^ FB (CD81^+^CD34^+^CD31^−^ cells) and stars, location of Igfbp5^+^ FB (CD81^−^CD34^+^CD31^−^ cells). **e** Arrows indicate location of (R1′) *Cxcl14*^+^Fgfr2^+^ FB (*Cxcl14*^+^*Fgfr2*^+^PDPN^+^ cells) and (R2′ and R3′) Fgfr2^+^ FB (*Fgfr2*^+^PDPN^+^ cells). **f** Arrows indicate location of CD90^+^ FB (PPARγ^+^CD34^+^CD31^−^ cells). **g** Arrows indicate location of Fgfr2^+^ FB (*Fgfr2*^+^PDPN^+^ cells). **h** Arrows indicate location of CD81^+^ FB (CD81^+^CD34^+^CD31^−^ cells). **d**–**h** Results are representative of 3 independent experiments. **i** Summary of FB subset location within the small intestinal and colonic lamina propria. SMC, smooth muscle cell. See also Supplementary Fig. 1.

Louvain clustering identified six small intestinal MSC clusters (Fig. 1a) and differential gene expression (DEG) analysis of these clusters identified pericytes, SMC and four FB clusters (Supplementary Fig. 1b). These were PDGFRα^hi^ FB, two PDGFRα^lo^CD34^hi^ clusters that could be distinguished based on their expression of *Cd81* (hereafter called CD81^+^ FB) and *Igfbp5* (hereafter called Igfbp5^+^ FB), and a PDGFRα^lo^CD34^lo^ cluster that expressed higher levels of *Fgfr2* (hereafter called Fgfr2^+^ FB) (Supplementary Fig. 1c). To determine how these clusters might relate to those identified in other, recently published scRNA-seq studies of small intestinal MSC^7,11^, DEGs from the previous MSC subsets were overlaid with our scRNA-seq dataset (Supplementary Fig. 1d). The MSC population termed “mural cells” by Hong et al.^11^ corresponded to our pericytes, while their FB subsets termed FB2, 3, 4 and 5 corresponded to our small intestinal Igfbp5^+^ FB, Fgfr2^+^ FB, CD81^+^ FB and PDGFRα^hi^ FB clusters, respectively (Supplementary Fig. 1d). The signature genes of FB1 identified by Hong et al. as activated FB based on their expression of *Junb* and *Fosb*, were expressed widely by several MSC subsets in our dataset (Supplementary Fig. 1d), indicating that this cluster represents a cell state rather than a MSC subset. Similar comparison with the MSC datasets generated by McCarthy et al.^7^ demonstrated that the PDGFRα^hi^ MSC subset they defined as “telocytes” corresponded to our PDGFRα^hi^ FB cluster, while the Lo-1 FB subset they named “trophocytes” corresponded to our CD81^+^ FB cluster and their Lo-2 FB subset encompassed both our Fgfr2^+^ and Igfbp5^+^ FB clusters (Supplementary Fig. 1d)^7^. Thus, our results confirm and extend recent findings and highlight the complexity of MSC subsets in the small intestinal LP, with there being substantial heterogeneity among PDGFRα^lo^ FB subsets in particular.

Louvain clustering also identified six MSC clusters in colon LP (Fig. 1b), which DEG analysis identified as pericytes, SMC, and four FB clusters (Supplementary Fig. 1e). These were PDGFRα^hi^ FB and three PDGFRα^lo^CD34^+^ clusters that could be distinguished based on their expression of *Cd81* (hereafter called CD81^+^ FB), *Cd90* (hereafter called CD90^+^ FB) or *Fgfr2* (hereafter called Fgfr2^+^ FB) (Supplementary Fig. 1f). To determine the relationship between the colonic and small intestinal MSC subsets, Pearson correlation analysis was performed based on the pseudo-bulk of overlapping variable genes between the two datasets. This showed that colonic pericytes, SMC, PDGFRα^hi^ FB, CD81^+^ FB correlate closely with their counterparts in the small intestine, that colonic CD90^+^ FB are most similar to small intestinal Igfbp5^+^ FB and that colonic Fgfr2^+^ FB correspond to both small intestinal Fgfr2^+^ and Igfbp5^+^ FB (Fig. 1c).

### FB subsets are located within distinct niches along the crypt-villus axis

There is increasing evidence that subsets of small intestinal FBs may occupy distinct anatomical niches that overlap with the WNT/BMP signaling gradient along the crypt-villus axis^7,10,27^. In line with a recent report^15^, we found that small intestinal CD34^+^ FB (which include CD81^+^ and Igfbp5^+^ FB) were located around crypts and in the submucosa, but were largely excluded from the villus core (Supplementary Fig. 1g). Of these, the CD81^+^ FB were located around CD31^+^ vessels close to and within the submucosa, with some locating close to crypts (Fig. 1d), consistent with recent reports^7,11,19^; the Igfbp5^+^ (CD34^+^CD81^−^) FB located around crypts (Fig. 1d). Conversely, PDGFRα^+^CD34^−^ FB (including both PDGFRα^hi^ FB and Fgfr2^+^ FB) were located directly underlying the epithelium and within the villus core (Supplementary Fig. 1g). PDGFRα^hi^ FB underlie the epithelium (Supplementary Fig. 1g)^7,9,11,27,34^ and RNA-scope analysis using an *Fgfr2* probe in combination with PDPN staining demonstrated that Fgfr2^+^ FB located throughout the villus core, suggesting they represent interstitial FB (Fig. 1e). Furthermore, RNA-scope analysis using a probe for *Cxcl14*, that was expressed by a large proportion of Fgfr2^+^ FB and a few PDGFRα^hi^ FB (Supplementary Fig. 1h), indicated that *Cxcl14* expressing FB preferentially located towards the villus tip (Fig. 1e).

As in the small intestine, colonic CD34^+^ FB subsets were located beneath and surrounding intestinal crypts, while PDGFRα^hi^ FB formed a thin layer directly underlying the epithelium and were concentrated at the top of crypts (Supplementary Fig. 1I). As colonic CD90^+^CD34^+^ FB expressed high levels of *Pparg* compared with other colonic FB subsets (Supplementary Fig. 1h), we stained for PPARγ to locate this subset, which was found at the base of colonic crypts (Fig. 1f). RNA-scope analysis using an *Fgfr2* probe in combination with PDPN demonstrated that Fgfr2^+^ FB localized preferentially between crypts (Fig. 1g), whereas the colonic CD81^+^ FB were located below the crypts and in the submucosa (Fig. 1h). For a summary of intestinal FB subset location see Fig. 1i. Collectively these results demonstrate that the FB subsets identified by scRNA-seq locate within distinct niches of the small and large intestine.

### Expression of epithelial support genes is largely conserved across FB subsets in the small intestine and colon

**Fig. 2:**
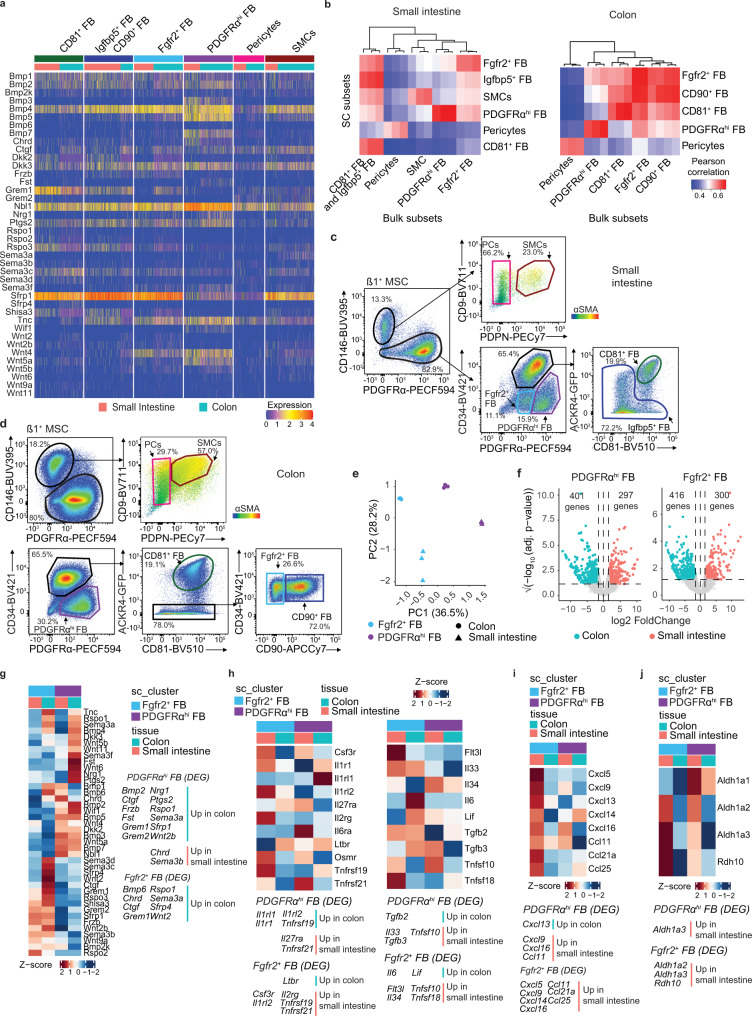
Despite similar FB subset composition, small intestinal and colonic FB display regional transcriptional specialization. **a** Heatmaps showing transcription levels (integrated data) of selected epithelial support genes by indicated MSC subsets. **b** Pearson correlations between averaged cluster expressions of Louvain clusters from scRNA-seq and bulk RNA-seq datasets based on 1937 (small intestine, left) and 1925 (colon, right) overlapping variable genes. Bulk RNA-seq data are from sorted MSC subsets from 3 independent experiments. Unsupervised hierarchical clustering indicates similarities of bulk RNA-seq subsets within each tissue. **c**, **d** Flow cytometric analysis of adult small intestinal (**c**) and colonic (**d**) Itgβ1^+^ MSCs from *Ackr4.GFP* mice. Representative staining from 2 experiments with 2–4 mice/experiment. Colored gates represent indicated MSC subsets. PCs pericytes, SMCs smooth muscle cells, FB fibroblast. **e** Principal component analysis (PCA) of bulk RNA-seq data from indicated sorted FB populations. Results are from 3 independent sorts/population. **f** Volcano plots showing differentially expressed genes (DEGs) between small intestinal and colonic PDGFRα^hi^ FB (left) and Fgfr2^+^ FB (right). Dotted horizontal line denotes significant adjusted *p* value (Benjamini–Hochberg correction) of 0.05, vertical dotted lines denote log_2_FC = 0 and the log_2_FC of ±1.5. **g**–**j** Heatmap representations of averaged transcription levels of indicated genes within sorted FB subsets. Data are averaged from 3 independent bulk RNA-seq datasets. **g** Epithelial support genes, **h** cytokines and cytokine receptors, **i** chemokines, **j** vitamin A metabolism. Gene lists for **i** were selected based on the epithelial support list in (**a**) while those in **h**–**j** were differentially expressed between either small intestinal and colonic PDGFRα^hi^ FB or between small intestinal and colonic Fgfr2^+^ FB. Identified DEG that are 1.5 < |log_2_FC| are listed to the right of (**g**) or below (**h**–**j**) the heatmaps and. See also Supplementary Fig. 2.

Recent studies have suggested a division of labor amongst small intestinal FB subsets in the production of epithelial support factors^5–10,16,17^ and we thus assessed the expression of such genes in our small intestinal and colonic FB datasets. Consistent with these studies^7,10^, small intestinal PDGFRα^hi^ FB were major producers of BMPs and this property was shared by colonic PDGFRα^hi^ FB (Fig. 2a). *Bmp3*, *Bmp5* and *Bmp7* expression was largely restricted to PDGFRα^hi^ FB, while expression of *Bmp1*, *Bmp2* and *Bmp4* was found more broadly among FB MSC subsets in both tissues (Fig. 2a). Both small intestinal and colonic PDGFRα^hi^ FB were also the dominant source of the non-canonical WNT ligands, *Wnt4*, *Wnt5a* and *Wnt5b*, although Fgfr2^+^ FB also expressed *Wnt4*, particularly in the small intestine (Fig. 2a). Consistent with previous results^7,9,11,12^, CD81^+^ FB were the major source of the BMP antagonist *Grem1* in the small intestine and this was also highly expressed by colonic CD81^+^ FB. However, in the colon, Fgfr2^+^ FB and CD90^+^ FB also expressed *Grem1* (Fig. 2a). Thus, the specialization of MSC subsets in their expression of epithelial support genes is largely conserved between the small intestine and colon.

### Small intestinal and colonic PDGFRα^hi^ FB and Fgfr2^+^ FB display regional transcriptional specificity

As described above, PDGFRα^hi^ and Fgfr2^+^ FB occupied very distinct locations within the mucosa, with the PDGFRα^hi^ subset being found immediately beneath the epithelium (Fig. 1i), where they are believed to regulate epithelial cell differentiation. However, little is known about the function of Fgfr2^+^ FB and to explore this, we examined our scRNA-seq datasets for surface markers that would allow us to identify and sort these cells for bulk RNA-seq analysis (Supplementary Fig. 2a). For the small intestine, pericytes were identified and sorted as PDGFRα^−^ESAM-1^+^PDPN^−^ cells, SMC as PDGFRα^−^ESAM-1^+^PDPN^+^ cells, PDGFRα^hi^ FB as ESAM-1^−^PDGFRα^hi^CD34^−^ cells, Fgfr2^+^ FB as ESAM-1^−^PDGFRα^int^CD34^−^ cells and CD34^+^ FB (including CD81^+^ and Igfbp5^+^ FB) as ESAM-1^−^PDGFRα^int^CD34^+^ cells. For the colon, pericytes were sorted as for small intestine, PDGFRα^hi^ FB were sorted as ESAM-1^−^PDPN^+^CD34^−^ cells, Fgfr2^+^ FB were sorted as ESAM-1^−^PDPN^hi^CD34^+^CD90^−^ cells, CD90^+^ FB as ESAM-1^−^CD34^+^PDPN^hi^CD90^+^ cells and CD81^+^ FB as ESAM-1^−^PDPN^int^CD34^+^CD90^−^ cells, based on the fact that colonic CD81^+^ FB express low levels of PDPN compared with the other CD34^+^ colonic FB subsets (Supplementary Fig. 2b). Correlation analysis of these bulk sorted intestinal FB subsets with the scRNA-seq data confirmed that flow cytometry and this staining panel could identify small intestinal and colonic FB subsets (Fig. 2b and Supplementary Fig 2c). This initial panel was then refined for use in subsequent flow cytometry-based analysis by including anti-CD81 to identify CD81^+^ FB directly, together with anti-CD146 (Fig. 2c, d), which can be used interchangeably with ESAM-1 (Supplementary Fig. 2d). CD81^+^ FB also expressed the atypical chemokine receptor, ACKR4, as assessed using *Ackr4*.GFP reporter mice (Fig. 2c, d)^19^, consistent with previous reports^7,9,11,19^ and our scRNA-seq analysis (Supplementary Fig. 1b).

PCA analysis of bulk sorted PDGFRα^hi^ FB and Fgfr2^+^ FB distinguished these subsets from one another in PC1, while PC2 separated small intestinal from colonic FB (Fig. 2e). Thus, PDGFRα^hi^ FB and Fgfr2^+^ FB have distinct transcriptional profiles, which are influenced by their anatomical location. To gain a broader understanding of the transcriptional differences between Fgfr2^+^ FB and PDGFRα^hi^ FB, we performed DEG analysis. This showed that Fgfr2^+^ FB expressed 403 genes at significantly higher levels than PDGFRα^hi^ FB, while PDGFRα^hi^ FB expressed 345 genes at higher levels (Supplementary Fig. 2e, for complete list see Supplementary Data File 1). Enrichr based gene ontology (GO) analysis (GO Biological Processes 2021^35,36^) demonstrated that compared with PDGFRα^hi^ FB, Fgfr2^+^ FB were significantly enriched in GO terms associated with extracellular matrix organization, complement activation, vasculogenesis, axonogenesis and several immune processes. The terms enriched in PDGFRα^hi^ FB included regulation of developmental processes, smooth muscle contraction and epithelial sheet morphogenesis (Supplementary Fig. 2f).

To determine how location impacts on the transcriptional profile of PDGFRα^hi^ and Fgfr2^+^ FB, we compared the gene expression profile of each subset from the small intestine and colon. Small intestinal PDGFRα^hi^ FB differed from their colonic counterparts in their transcription of 698 genes (Fig. 2f, Supplementary Data File 2), while small intestinal and colonic Fgfr2^+^ FB differed in their transcription of 716 genes (Fig. 2f, Supplementary Data file 2). Of these, 149 genes were differentially expressed in both FB subsets (Supplementary Fig. 2g and Supplementary Data File 3). These included numerous Hox genes (Supplementary Fig. 2h), consistent with their role in regional patterning of the intestine^37^. Enrichr based GO analysis demonstrated that colonic PDGFRα^hi^ FB were significantly enriched in pathways associated with epithelial support and wound healing compared with small intestinal PDGFRα^hi^ FB, while the latter were enriched in pathways associated with responses to TGFβ, ion transport and anterior/posterior pattern specification (Supplementary Fig. 2I). This analysis further showed colonic Fgfr2^+^ FB to be enriched in pathways associated with enhanced transcription, extracellular matrix organization and epithelial support, while small intestinal Fgfr2^+^ FB were enriched in pathways that included response to IFNγ, defense response to protozoa as well as anterior/posterior pattern specification.

Closer analysis of DEGs revealed that PDGFRα^hi^ FB and Fgfr2^+^ FB expressed a distinct array of epithelial support genes, irrespective of their location along the length of the intestine (Fig. 2g). Notably, many of these genes were expressed at significantly higher levels in colonic subsets compared with their small intestinal counterparts (Fig. 2g). PDGFRα^hi^ FB and Fgfr2^+^ FB also expressed a wide range of immunologically relevant genes in both a subset- and tissue-specific manner (Fig. 2h–j). These included several cytokine and cytokine receptors (Fig. 2h), while small intestinal but not colon Fgfr2^+^ FB expressed many chemokine genes (Fig. 2i). Both subsets of small intestinal FB also expressed enzymes implicated in the metabolism and generation of retinoic acid (Fig. 2j), a major regulator of small intestinal immune responses. Importantly, these bulk RNA-seq expression profiles largely overlapped with our scRNA-seq datasets (Supplementary Fig. 2j), although data from the latter was, as expected, sparser and at lower expression levels generally. Collectively, these results highlight the unique transcriptional profiles of intestinal PDGFRα^hi^ FB and Fgfr2^+^ FB and show that these vary depending on location along the length of the intestine.

### Precursors in E12.5 intestine can give rise to all adult intestinal MSC subsets

**Fig. 3:**
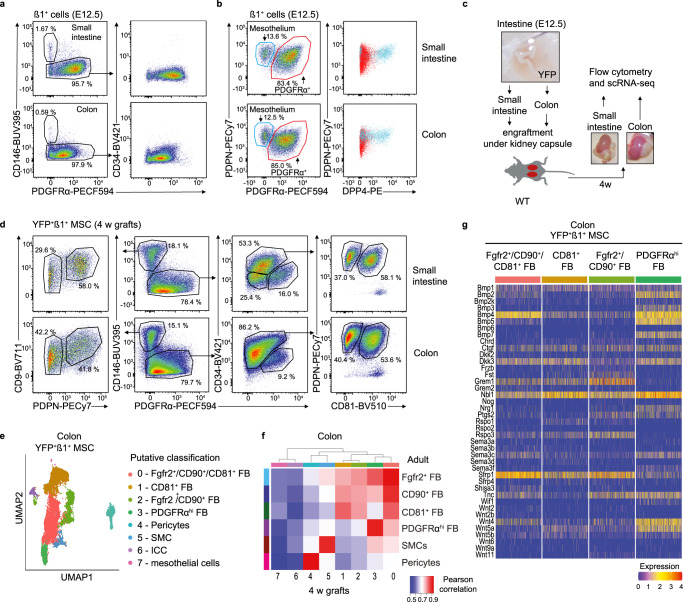
Adult intestinal MSC subsets derive from intestinal precursors present in E12.5 intestine. **a**, **b** Flow cytometric analysis of Itgβ1^+^ MSCs isolated from indicated organs on embryonic day (E) 12.5. **b** Right hand plots show expression of DPP4 (CD26) on gated PDPN^+^PDGFRα^−^ (blue) and PDPN^+^PDGFRα^+^ (red) cells from plots on left. Data are representative of **a** 4 experiments with 2–8 embryos/experiment, or **b** 3 experiments with 6–8 individual embryos. **c** Workflow of transplantation of E12.5 intestine from YFP^+^ mice under the kidney capsule of WT recipients. **d** Flow cytometric analysis of YFP^+^Itgβ1^+^ MSC in intestinal grafts 4 weeks after transplantation. Results are representative of 2 experiments with 4 (small intestine) or 2–3 (colon) grafts/experiment. **e** UMAP dimensionality reduction of scRNA-seq data colored by Louvain clustering from FACS purified YFP^+^Itgβ1^+^ MSC isolated from colonic grafts 4 weeks after transplantation. Data are from 8624 single cells from 3 pooled colonic grafts with an average of 2223 genes/cell. **f** Pearson correlations of averaged gene expression in colonic graft and adult colon MSC clusters based on 1486 overlapping variable genes. **g** Heatmap showing transcription levels (integrated data) of selected epithelial support genes within the putative corresponding FB clusters identified in (**e**). See also Supplementary Fig. 3.

While adult small intestinal and colonic LP contains multiple phenotypically, transcriptionally and spatially distinct MSC subsets, the developmental relationship between these subsets and whether all derive from similar precursors remain unclear^20,31,32^. To address this, we first investigated which MSC might be present in the small intestine and colon of E12.5 embryos by flow cytometry (Fig. 3a and Supplementary Fig. 3a). In contrast to adult mice (Fig. 2c, d), E12.5 small intestinal and colonic Itgβ1^+^ cells consisted mostly of PDGFRα^+^CD34^−^ MSCs. However there was also a small subset of PDGFRα^−^ cells that expressed dipeptidyl peptidase-4 (DPP4, CD26) and PDPN (Fig. 3a, b), markers of mesothelial cells^38^ that have previously been suggested to be a source of precursors for some intestinal FB and SMC in the developing embryo^31,32,39^. To address whether these populations could give rise to the MSC subsets found in the adult intestine, small and large intestine from E12.5 embryos ubiquitously expressing EYFP were transplanted under the kidney capsule of adult wildtype recipient mice (Fig. 3c). As expected^40–42^, small intestinal and colonic grafts had increased markedly in size by 4–6 weeks post transplantation (Fig. 3c) and contained mucosa that histologically resembled adult small intestine and colon, respectively (Supplementary Fig. 3b). To assess the phenotypic diversity of graft-derived MSC, small intestinal and colonic grafts were isolated 4 weeks after transplantation, digested, and the expression of MSC subset markers on graft-derived (YFP^+^) Itgβ1^+^ MSC assessed by flow cytometry (Fig. 3d and Supplementary Fig. 3c). Both small intestinal and colonic grafts contained putative populations of graft-derived SMC (CD146^+^PDGFRα^−^PDPN^+^), pericytes (CD146^+^PDGFRα^−^PDPN^−^), PDGFRα^hi^ FB (CD146^−^CD34^−/lo^PDGFRα^hi^), CD81^+^ FB (CD146^−^PDGFRα^lo^CD81^+^CD34^+^) and CD81^−^CD34^+^ FB (CD146^−^PDGFRα^lo^CD34^+^CD81^−^) (Fig. 3d). To confirm the presence of these MSC subsets in the grafts, YFP^+^Itgβ1^+^ MSC were sorted from grafted colon and subjected to scRNA-seq (Supplementary Fig. 3c). UMAP dimensionality reduction and Louvain clustering identified eight clusters (Fig. 3e), two of which (clusters 6 and 7) were identified as ICC and mesothelial cells, respectively^43–45^ (Supplementary Fig. 3d). These clusters were not part of our adult MSC datasets, as ICC were removed bioinformatically, while the mesothelium was removed together with the muscularis externa during tissue processing. Pearson correlation analysis based on the pseudo-bulk of overlapping variable genes identified cluster 3 as being similar to adult PDGFRα^hi^ FB, cluster 4 as pericytes and cluster 5 as SMC (Fig. 3e, f). The remaining three clusters (clusters 0–2) were more closely related to the three adult CD34^+^ FB subsets, with cluster 1 correlating most closely to CD81^+^ FB, cluster 0 most closely to Fgfr2^+^/CD90^+^ FB and cluster 2 showing overlap with all three adult CD34^+^ FB subsets (Fig. 3f). Furthermore, the distinct expression of epithelial support genes by each of the four FB subsets largely overlapped with the pattern seen in adult intestine (Fig. 3g and 2a). Collectively, these results suggest that MSC precursors present in E12.5 intestine can give rise to all adult intestinal MSC subsets.

### Adult intestinal MSC derive from *Gli1*^*+*^ embryonic precursors

**Fig. 4:**
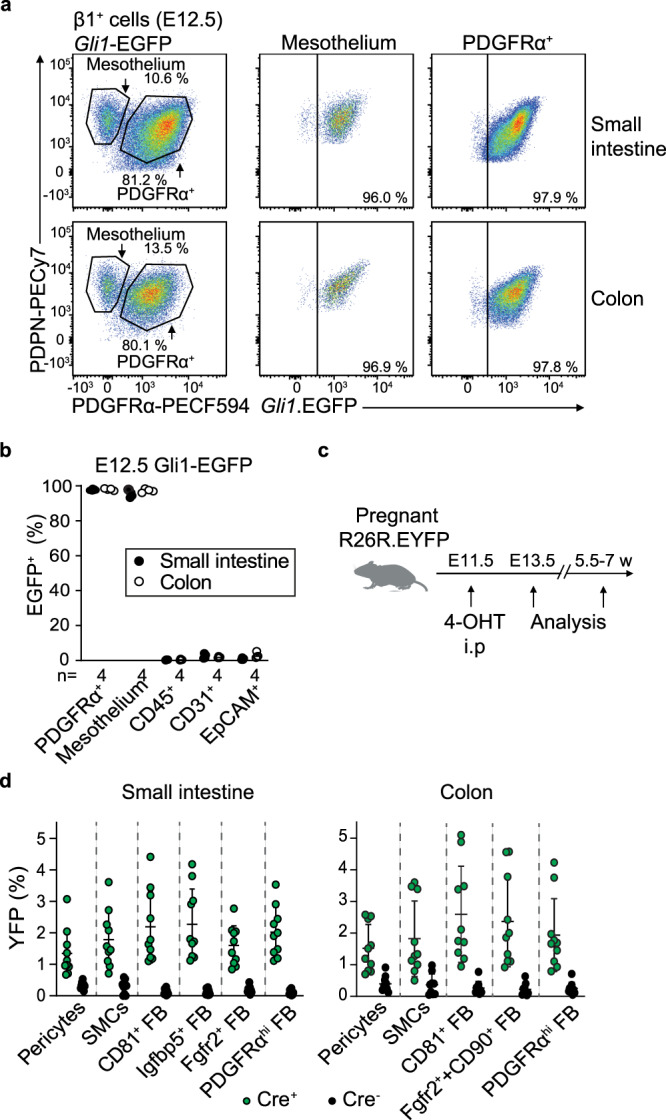
Adult intestinal MSC derive from *Gli1*^*+*^ embryonic precursors. **a** Representative flow cytometric analysis and **b** proportions of indicated cells expressing EGFP in the small intestine and colon of embryonic E12.5 *Gli1*-EGFP mice. Results are from 8 individual embryos, with each circle representing an individual embryo. **c** Workflow of lineage-tracing experiments. *R26R*.EYFP females were mated overnight with *Gli1*.CreERT2^+/−^ males and pregnant dams injected i.p. with 4-hydroxytamoxifen (4-OHT) at E11.5. **d** Proportions of indicated MSC subset expressing YFP in small intestine and colon of 5.5–7-week-old *Gli1*.CreERT2^+/−^.*R26R*.EYFP (Cre^+^) and *R26R*.EYFP (Cre^−^) littermates. Results are pooled from 4 independent experiments with 10 Cre^+^ mice and 11 Cre^−^ mice. Each circle represents an individual mouse. Bars represent the means and SD. See also Supplementary Fig. 4. Source data are provided as a Source Data file.

To explore further the origin of adult MSC, we next lineage-traced E12.5 MSC and mesothelium into adulthood. GLI1 is a transcription factor induced by active hedgehog-signaling and is expressed by MSC in multiple organs^46^, including early embryonic and adult intestinal MSC^6,47,48^. Consistent with this, a large proportion of each of the MSC subsets present in the adult small intestine and colon of *Gli1*-EGFP mice expressed *EGFP* (Supplementary Fig. 4a). Further, while *Gli1* transcripts were sparse in our scRNA-seq datasets, they were present across all adult intestinal MSC subsets (Supplementary Fig. 4b). In E12.5 *Gli1*-EGFP embryos, small intestinal and colonic PDGFRα^+^ MSC and PDGFRα^−^PDPN^+^ mesothelial cells expressed EGFP, whereas intestinal epithelial, endothelial and CD45^+^ cells did not (Fig. 4a, b). Again, while *Gli1* transcripts were sparse in our embryonic scRNA-seq datasets, they were present in both embryonic mesothelial cells and PDGFRα^+^MSC (Supplementary Fig. 4c). To lineage-trace *Gli1*-expressing cells into adulthood, female *R26R*.EYFP mice^49^ were mated with *Gli1*-Cre.ERT2 males expressing the estrogen receptor (ERT2) under control of *Gli1*-Cre, and pregnant dams injected i.p with 4-hydroxytamoxifen (4-OHT) at E11.5 (Fig. 4c). Two days later, YFP expression had been induced in a small but consistent proportion of Itgβ1^+^PDGFRα^+^ MSC and mesothelial cells in the small intestine and colon of *Gli1*-CreERT2^+/−^.*R26R*.EYFP embryos, but not in Cre^−^ embryos (Supplementary Fig. 4d, e). Labeling was not observed in intestinal epithelial, endothelial or CD45^+^ immune cells of *Gli1*-CreERT2^+/−^.*R26R*.EYFP mice and thus was specific to intestinal MSC and mesothelial cells (Supplementary Fig. 4d, e). Five-seven weeks after birth, similar proportions of YFP-expressing cells were detected in all mature MSC subsets in both the small intestine and colon (Fig. 4d). Given the similar proportions of intestinal MSC that are labeled in E13.5 embryos and adult mice, these results suggest that the majority of adult intestinal MSC derives from *Gli1*^*+*^ cells present in the E12.5 intestine.

### Trajectory analysis indicates that adult intestinal FB subsets develop in a linear fashion from embryonic *Gli1*^*+*^ precursors

**Fig. 5:**
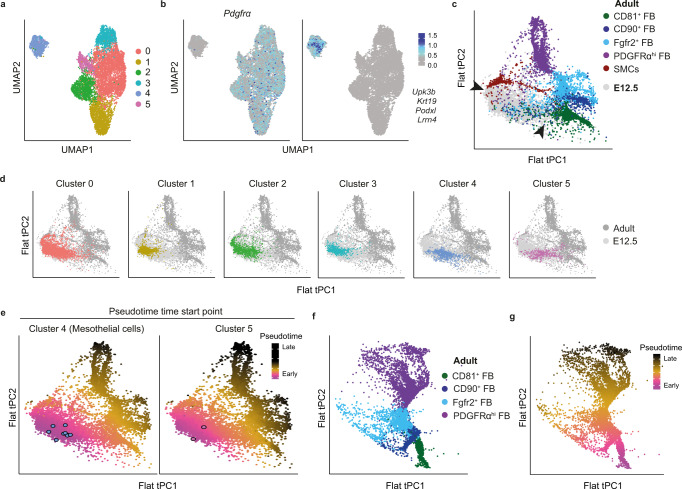
Trajectory analysis indicates that adult intestinal FB subsets develop in a linear fashion from embryonic *Gli1*^*+*^ precursors. **a** UMAP dimensionality reduction of scRNA-seq data colored by Louvain clustering from FACS purified Itgβ1^+^ MSC from the colon of embryonic day E12.5 mice. Data are from 9632 single cells from 2 pooled experiments using 3–5 embryonic colons/experiment, with an average of 2521 genes/cell. **b** UMAP of E12.5 large intestinal Itgβ1^+^ MSC overlaid with expression of *Pdgfra* or the indicated mesothelium associated genes. **c**, **d** tSPACE principal component analysis (tPC1–3) projection of pooled adult colonic and E12.5 large intestinal MSC embedded into 2D space. **c** Adult clusters are color coded as indicated, while E12.5 clusters are in gray. Arrow heads indicate connections between embryonic and adult clusters. **d** Location of indicated E12.5 clusters on tPC projections of adult (dark gray) and embryonic (light gray) MSC. **e** Pseudotime analysis using averaged values of the 9 trajectory starting points (highlighted blue cells) in embryonic Cluster 4 (mesothelial cells, left panel) or 2 trajectory starting points (highlighted pink cells) in embryonic Cluster 5 (right panel) superimposed on the tPC projection. **f** tSPACE projections of indicated adult colonic MSC in tPC1–3 embedded into 2D space and **g** pseudotime analysis superimposed on (**f**) using averaged values of the 215 trajectories starting in CD81^+^ FB. See also Supplementary Fig. 5.

To gain further insights into the relationship between embryonic intestinal *Gli1*^*+*^ cells and adult intestinal MSC subsets, scRNA-seq was performed on fluorescently activated cell sorted Itgβ1^+^ MSC from the colon of E12.5 embryos. Louvain clustering identified six clusters (Fig. 5a), 5 of which (clusters 0–3 and 5) expressed *Pdgfra* (Fig. 5b) and represented embryonic FB and one of which, cluster 4, was identified as mesothelial cells due to its expression of mesothelial associated markers^44,45^ (Fig. 5b). Consistent with our flow cytometric analysis (Fig. 3b), this cluster also expressed transcripts for *Dpp4* and *Pdpn*, but lacked expression of *Pdgfra* (Fig. 5b, Supplementary Fig. 5a). The mesothelial associated gene Wilms tumor 1 (*Wt1*)^31,39^, whose expression is maintained in cells that have recently undergone epithelial to mesenchymal transition (EMT)^50^, was expressed by embryonic mesothelial cells but also by cells within FB cluster 5 (Supplementary Fig. 5b). This indication that FB cluster 5 may represent a population of FB that have recently undergone EMT is supported by the fact that both embryonic mesothelial cells and FB cluster 5 expressed several genes associated with FB progenitors^51–56^ (Supplementary Fig. 5c). To assess the relationship between embryonic and adult MSC subsets, the embryonic and adult colonic datasets were integrated and tSPACE^57^ trajectory analysis was performed on MAGIC imputed sets of variable genes, as described previously^58,59^. Pericytes were removed from this analysis, as too few of these cells were present in the adult dataset to generate meaningful conclusions. Three-dimensional visualization of tSPACE principal components (tPC) 1–3 demonstrated that embryonic cells clustered together and away from adult MSC subsets (Fig. 5c). Nevertheless, two distinct connections were observed between embryonic and adult colonic MSC (Fig. 5c, arrow heads), one between embryonic clusters and adult SMC, and the second between embryonic clusters and adult CD81^+^ FB and to a lesser extent adult CD90^+^ FB (Fig. 5c). Mapping the location of the individual embryonic clusters within the tSPACE projection suggested that the embryonic FB that connected directly with adult SMC were enriched in clusters 0 and 2, whereas the embryonic FB that connected directly with adult CD81^+^ FB were enriched in cluster 5 (Fig. 5d). The location of mesothelial cluster 4 and cluster 5 within the tSPACE projection was largely overlapping (Fig. 5d), suggesting direct trajectory connections between these populations. As intestinal mesothelial cells at this stage of embryonic gut development undergo EMT and give rise to some intestinal FB and SMC^31,32,39^, while both cluster 5 FB and the embryonic mesothelium shared features of FB progenitors (Supplementary Fig. 5c), we selected these clusters as tSPACE trajectory starting points for pseudotime analysis (Fig. 5e). Both starting points indicated similar pseudotime trajectories towards either adult SMC or adult CD81^+^ FB (Fig. 5e). Collectively, this bioinformatic based analysis indicates that *Gli1*^+^ embryonic precursors may give rise to both SMC and FB in the adult intestine via distinct embryonic intermediates.

Interestingly, rather than branching directly and separately into the various FB subsets, the only direct connection of adult CD81^+^ FB was to CD90^+^ FB and these then connected to Fgfr2^+^ FB and finally to PDGFRα^hi^ FB (Fig. 5c); tSPACE analysis of adult FB alone showed a similar linear connection between adult FB subsets (Fig. 5f). This finding is consistent with a recent FB atlas of multiple mouse tissues published by Buechler et al. that identified two universal subtypes of FB, *Pi16*^+^ FB and *Col15a1*^+^ FB, with the *Pi16*^+^ FB containing precursors that are capable of giving rise to all tissue-specific FB subsets via a *Col15a1*^+^ intermediate^60^. Overlay of DEG from the FB clusters defined by Buechler et al. onto our adult colonic FB datasets demonstrated that the *Pi16*^+^, *Col15α1*^+^, *Fbln1*^+^ and *Bmp*4^+^ FB clusters broadly overlapped with our colonic CD81^*+*^, CD90^*+*^, Fgfr2^*+*^ and PDGFRα^hi^ FB subsets, respectively (Supplementary Fig. 5d). We thus used CD81^*+*^ FB as the starting population for a new pseudotime analysis of adult colonic FB, which indicated a linear trajectory from adult CD81^+^ FB via CD90^+^ FB and Fgfr2^+^ FB to PDGFRα^hi^ FB (Fig. 5g). Collectively, this bioinformatic analysis suggests that adult intestinal FB arise sequentially from CD81^+^ FB.

### Colonic PDGFRα^hi^ FB may arise directly from Fgfr2^+^ FB and consist of three transcriptionally distinct clusters

**Fig. 6:**
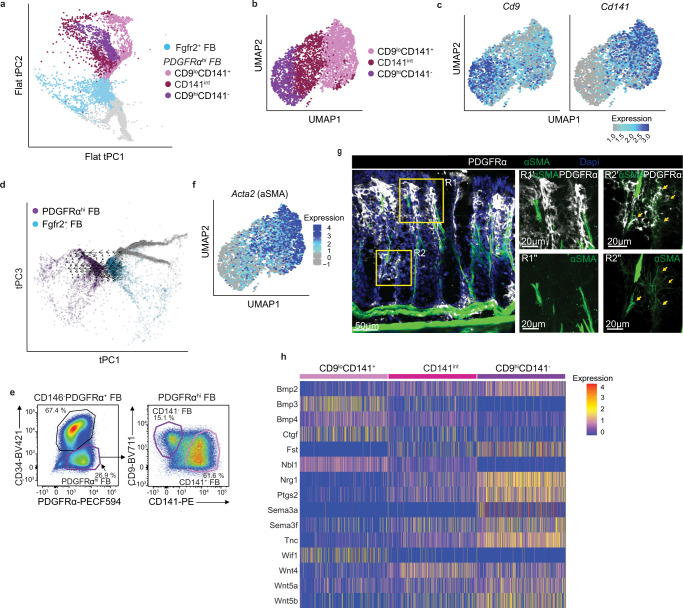
Colonic PDGFRα^hi^ FB may arise directly from Fgfr2^+^ FB and consist of three transcriptionally distinct clusters. **a** tSPACE projection of colonic MSC in tPC1–3 embedded into 2D space, highlighting Fgfr2^+^ FB, together with three PDGFRα^hi^ FB clusters. **b** UMAP dimensionality reduction of re-clustered colonic PDGFRα^hi^ FB depicting the three indicated subclusters and **c** expression *of Cd9* (left panel) and *Cd141* (right panel) overlaid onto the UMAP in (**b**). **d** tSPACE projection of adult colonic MSC in tPC1 and 3, highlighting Fgfr2^+^ FB and PDGFRα^hi^ FB overlaid with RNA velocity estimates. **e** Representative flow cytometric analysis of CD9 and CD141 expression by colonic PDGFRα^hi^ FB. Representative plots from 2 experiments with 3 mice/experiment. **f** *Acta2* (αSMA) gene expression projected onto UMAP of colonic PDGFRα^hi^ FB in (**b**). **g** Immunohistochemical staining of colonic tissue for indicated antigens. R1′^,^″ and R2′^,^″ represent magnifications of R1 and R2 quadrants (yellow squares) on left image. Results are representative stains from 3 experiments with 3 mice/experiment. Arrows indicate αSMA^+^PDGFRα^hi^ FB. **h** Heatmap showing transcription levels (integrated data) of significantly (*p* < 0.05) differentially expressed epithelial support genes between the PDGFRα^hi^ FB clusters. See also Supplementary Fig. 6.

Subepithelial PDGFRα^hi^ FB have been described as both “telocytes” and αSMA expressing “myofibroblasts”^5,7,10,12,27^, but whether all these cells represent the same, distinct, or partially overlapping populations remains unclear. Furthermore, as PDGFRα^hi^ FB are believed to support epithelial cell function in anatomically distinct compartments, the stem cell niche^5,10^ and in the region where epithelial cells differentiate^5,7,9,27^, there may be further sub-specialization of these FB depending on their location. Consistent with additional heterogeneity, our tSPACE analysis suggested that PDGFRα^hi^ FB diverged along three distinct trajectories (Fig. 5f, g) and to assess whether these trajectories reflected potential heterogeneity among PDGFRα^hi^ FB, we isolated the PDGFRα^hi^ FB scRNA-seq data and re-clustered the cells at higher resolution. This identified three subclusters, each of which aligned along a distinct trajectory branch when mapping back to the adult tPC projection (Fig. 6a, b). These clusters could be distinguished based on their expression of *Cd9* and *Cd141* (thrombomodulin (Thbd)), into *Cd9*^hi^*Cd141*^−^, *Cd9*^lo^*Cd141*^+^ and *Cd141*^int^ cells (Fig. 6b, c), all of which expressed the “telocyte” marker, *Fox1l* (Supplementary Fig. 6a)^10^. RNA velocity analysis^61^ suggested that all three clusters originated from Fgfr2^+^ FB (Fig. 6d). Similar CD9^hi^CD141^−^, CD9^lo^CD141^+^ and CD9^−^CD141^+/int^ subsets of colonic PDGFRα^hi^ FB could be identified by flow cytometry (Fig. 6e). Analysis of the top DEG between these populations demonstrated that *Cd9*^hi^*Cd141*^−^ cells expressed the highest levels of *Nrg1*, *Fgf7, Il1rl1* (ST2 (IL33 receptor)) and *Ptgs2*, while the *Cd9*^lo^*Cd141*^+^ cells expressed high levels of fibrosis-associated *Aspn* (Asporin), *Il11ra1* and *Cxcl12*, and the *Cd141*^int^ cells expressed high levels of *Cxcl10*, *Ly6c1*, *Adamdec1*, *Wnt4a* and *Plpp3* (Supplementary Fig. 6b). Interestingly, only CD9^lo^CD141^+^ cells, and to a lesser extent the CD141^int^ cells, expressed mRNA and protein for αSMA (Fig. 6f and Supplementary Fig. 6c), a marker used to define subepithelial “myofibroblasts”. Immunohistochemical staining for PDGFRα and αSMA showed that αSMA^+^PDGFRα^hi^ cells localized preferentially to the isthmus area just above colonic crypts, while αSMA^−^PDGFRα^hi^ cells aligned directly underneath the epithelium at the top and bottom of crypts (Fig. 6g). The CD9^lo^CD141^+^, CD9^hi^CD141^−^ and CD141^int^ FB also differentially expressed several epithelial support genes (Fig. 6h), suggesting that these populations may play distinct roles in supporting the epithelium at different stages of its development. Thus, adult colonic subepithelial PDGFRα^hi^ FB consist of spatially and transcriptionally distinct clusters, only a proportion of which express the myofibroblast marker αSMA.

## Discussion

Recent studies have demonstrated considerable heterogeneity within the intestinal LP MSC compartment^5–7,11,12,25,27^ and suggested non-redundant roles for MSC subsets in intestinal homeostasis^7–9,11,27^, inflammation^5,26,62^ and cancer^25^. In addition, analysis of pooled scRNA-seq datasets from different studies suggest that MSC subset composition is similar in the small intestine and colon and in mouse and human intestine^12^. Here, we confirm and extend these findings by showing that the location of intestinal MSC subsets and their expression of epithelial support genes is largely conserved between the small intestine and colon. Nevertheless, by generating flow cytometry panels for identifying and sorting small intestinal and colonic MSC subsets from the same mice, we show significant differences in the transcriptional profile of equivalent MSC subsets between the small intestine and colon, suggesting an important role for the local environment in regulating niche specific MSC functions. Furthermore, by using a combination of transplantation and lineage-tracing approaches, scRNA-seq and bioinformatics analysis, we provide important insights into the ontogeny and developmental trajectories of intestinal MSC subsets, and demonstrate that these cells derive from *Gli1*^*+*^ precursors present in the E12.5 intestine.

Consistent with previous reports^7,10,11,34,63^, we found that PDGFRα^hi^ FB lie directly underneath the epithelium in both the small and large intestine, while CD81^+^ FB were present in the submucosa and surrounding larger vessels deep in the mucosa, in proximity to the epithelial stem cell niche. In contrast, the location of intestinal CD81^−^PDGFRα^lo^ FB has not yet been fully elucidated. Our scRNA-seq analysis demonstrated that CD81^−^PDGFRα^lo^ FB in both the small intestine and colon consisted of two populations; in addition to the Fgfr2^+^ FB subset found in both the small intestine and colon, there was a CD90^+^ FB subset in the colon and an Igfbp5^+^ subset in the small intestine. We found that the latter two transcriptionally related FB subsets located primarily near the base of colonic crypts indicating that they primarily represent peri-cryptal populations of FB. In contrast, Fgfr2^+^ FB were located along the length of the villus core in the small intestine and within the isthmus between colonic crypts. These findings are consistent with studies using *Fgfr2-*mCherry reporter mice^25^ and suggest the Fgfr2^+^ FB primarily represent interstitial FB^25^. Collectively, these studies of intestinal FB subsets confirm and extend previous work, highlighting their distinct transcriptional profiles and complex spatial organization within the mucosa.

To gain additional insights into intestinal FB subsets, as well as tissue-specific differences in FB function, we generated and validated a flow cytometry panel that allowed us to sort and perform bulk RNA-seq analysis on individual FB subsets. We focused our downstream analysis on PDGFRα^hi^ FB and Fgfr2^+^ FB, the former because of their subepithelial location and the known differences in the composition of the epithelium between the small intestine and colon^1^, and the latter because there is limited knowledge of their function^11,25^. GO analysis identified multiple transcription pathways that were enriched in Fgfr2^+^ FB compared with PDGFRα^hi^ FB and suggested that these cells play a more dominant role in extracellular matrix organization, immune response regulation, as well as the regulation of endothelial cell and neuronal growth, consistent with their interstitial location. In contrast, PDGFRα^hi^ FB were enriched in pathways associated with epithelial sheet morphogenesis and development, as well as smooth muscle contraction, the latter consistent with the observation that a proportion of these cells express αSMA.

We also observed a marked difference in the transcriptional profile of PDGFRα^hi^ FB and Fgfr2^+^ FB depending on whether they derived from the small intestine or colon. Both PDGFRα^hi^ and Fgfr2^+^ FB expressed higher levels of many epithelial support genes in the colon compared with the small intestine, suggesting there may be a greater need for FB support of epithelial integrity in the colon compared with the small intestine. Consistent with this, WNT secretion by *Gli1*-expressing MSC is essential for homeostasis of the colonic epithelium^6,9,16^, but this is less important in the small intestine, where Paneth cells represent a major source of WNTs^18,64^. While few pathways were selectively upregulated in small intestinal FB subsets, both small intestinal Fgfr2^+^ FB and PDGFRα^hi^ FB subsets were enriched in the GO term for “anterior/posterior pattern specification”, expressing selected Hox genes. Both subsets in the small intestine also expressed higher levels of several genes encoding enzymes involved in vitamin A metabolism, consistent with previous findings that some small intestinal FB display aldehyde dehydrogenase activity and that there is increased retinoic acid receptor signaling in the small intestine compared with the large intestine^21,65^. Small intestinal Fgfr2^+^ FB were also enriched in the GO terms “response to IFNγ”and “defense response to protozoa” and expressed higher levels of genes encoding chemokines, cytokines and cytokine receptors than their colonic counterparts, suggesting a more prominent immunological role for these cells in the steady state small intestine compared with the colon. Collectively, these findings indicate that the local microenvironment plays a crucial role in regulating the transcriptional profile and specialization of intestinal FB in different regions of the intestine. The nature of these environmental factors and their importance in local tissue homeostasis awaits further study.

The mesothelium is an epithelial monolayer that lines body cavities and internal organs, including the serosal surface of the intestine^66^. While mesothelial cells are not thought to undergo EMT in the steady state postnatal intestine^39^, tissue injury can induce EMT in which mesothelium gives rise to both SMC and FB in a number of tissues including the intestine^30,32,67^. In contrast to the postnatal intestine, the intestinal mesothelium has been shown to undergo EMT in early embryonic development and give rise to SMC in the intestinal muscle layers and vasculature^31,39^, as well as an uncharacterized FB in the outer serosa of the intestine^32^. Whether mesothelium or other precursors present in the early embryonic intestine contribute to the MSC subsets present in the adult small intestine and colon LP has remained unclear. Here we used intestinal transplantation and lineage-tracing approaches to demonstrate that all adult small intestine and colon LP MSC subsets derive from *Gli1*-expressing progenitors present in the E12.5 intestine. At that time point, *Gli1* expression was restricted to mesothelial cells and a population of PDGFRα^+^ FB. scRNA-seq analysis showed that both mesothelial cells and a minor cluster of cells within the PDGFRα^+^ FB population expressed markers previously associated with FB progenitors, with tSPACE analysis suggesting a direct trajectory connection between mesothelial cells and cluster 5. Collectively this suggests that *Gli1*^*+*^ mesothelial cells in E12.5 intestine may be the source of all adult LP FB subsets, although this remains to be proven directly and we cannot rule out the possibility that *Gli1*^*+*^ cells of non-mesothelial origin within embryonic cluster 5 also contribute to adult LP FB generation. Of note, although *Wt1*-CreERT2 mice have been used previously to lineage-trace cells derived from mesothelium^39^, they would not be appropriate for discriminating between mesothelial cells and cells within cluster 5 in the E12.5 intestine, as both expressed *Wt1*. Thus, this issue remains to be explored using more precise approaches.

Our tSPACE analysis revealed direct connections between embryonic MSC clusters and adult CD81^+^ FB, suggesting that all adult intestinal FB subsets arise via CD81^+^ FB, rather than from distinct populations of embryonic intermediates. Consistent with this possibility, we found intestinal CD81^+^ FB to be located at the base of the mucosa in the adventitia surrounding larger vessels, an anatomical niche that contains MSC progenitors in other tissues^46,68–73^. In addition, our intestinal CD81^+^ FB were transcriptionally related to the *Pi16*^+^ FB recently reported to be an adventitial FB population capable of giving rise to additional FB subsets in a variety of tissues^60^. Finally, lineage-tracing experiments have shown that intestinal *Grem1*^*+*^ FB, which are equivalent to CD81^+^ FB, give rise to subepithelial FB along the entire crypt-villus axis^55^. Collectively, these findings suggest that CD81^+^ FB act as precursors of FB subsets in the adult intestine.

While early bioinformatics based trajectory studies of intestinal FB suggested a bifurcation downstream from CD81^+^ FB^5^, our tSPACE, pseudotime and Velocity analyses suggested that there was a linear progression of colonic CD81^+^ FB to adult CD90^+^ FB, then to Fgfr2^+^ FB and finally to PDGFRα^hi^ FB. Again, this conclusion is consistent with the recent work on *Pi16*^+^ FB precursors, which have been shown to give rise first to *Col15α1*^+^ FB (transcriptionally related to our CD90^+^ FB) and then to tissue-specific FB clusters that in the intestine included *Fbln1*^+^ FB (related to our Fgfr2^+^ FB) and finally to *Bmp4*^+^ FB (related to our PDGFRα^hi^ FB)^60^. Interestingly, the trajectory from CD81^+^ FB also correlated with the basal to apical localization of the downstream FB subsets in the colonic LP, indicating that this process may be driven by factors present in distinct microenvironmental niches. Notably, FB populations turn over slowly in the steady state adult intestine^5,27,55^ and our results do not exclude the possibility that under these conditions individual FB subsets may self-maintain without input from upstream precursors.

Recent scRNA-seq studies have suggested that colonic subepithelial PDGFRα^hi^ FB are heterogeneous^5,25,27^ and here we found that PDGFRα^hi^ FB diverged into 3 clusters, which we could define as CD9^lo^CD141^+^, CD9^hi^CD141^−^ and CD141^int^ FB. The CD9^lo^CD141^+^ FB are likely related to the PDGFRα^hi^ FB subcluster S2a defined by Kinchen et al., as they expressed high levels of *Cxcl12*, while CD9^hi^CD141^−^ FB expressed high levels of *Nrg1* and so are likely related to the PDGFRα^hi^ FB subcluster S2b^5^. Although all three clusters expressed the telocyte marker *Foxl1*^10^, CD9^lo^CD141^+^ FB and to a lesser extent CD141^int^ FB, expressed *Acta2*, coding for αSMA, a marker often associated with myofibroblasts. αSMA^+^PDGFRα^hi^ FB were located directly underneath the epithelium approximately halfway up colonic crypts, suggesting that CD9^lo^CD141^+^ and CD9^hi^CD141^−^ subepithelial FB localize within distinct regions along the crypt axis. Of the three PDGFRα^hi^ FB clusters, CD9^lo^CD141^+^ FB expressed the highest levels of the WNT antagonists *Wif1*, *Bmp3* and *Bmp4*. Therefore we speculate that the location of CD9^lo^CD141^+^ FB halfway up colonic crypts allows them to promote the terminal differentiation of epithelial cells as they migrate up the crypt^74^. In contrast, CD9^hi^CD141^−^ FB expressed high levels of top of crypt-associated non-canonical *Wnt4, Wnt5a* and *Wnt5b*^75,76^ and *Tenascin C (Tnc)*^77,78^ and base of crypt-associated *Ptgs2* (the gene encoding COX-2)^15,25^ and *Sema3a*^16^. Thus, our findings indicate that each of the three PDGFRα^hi^ FB subsets may play distinct roles in colonic epithelial homeostasis.

In conclusion, our study provides a comprehensive mapping of intestinal MSC diversity, location and epithelial support function and highlights a central role for location along the intestinal length in regulating the transcriptional profile and functional specialization of intestinal FB. We also show that all adult MSC derive from *Gli1*-expressing embryonic precursors and propose these to be of mesothelial origin. Our data further suggest that there is a linear developmental relationship between adult FB subsets that culminates in the development of a heterogeneous group of subepithelial PDGFRα^hi^ FB. Together our findings provide key insights into MSC diversity, development, function and interrelationships with relevance to intestinal development and homeostasis.

## Methods

### Mice and ethical statements

*Gli1*^tm3(cre/ERT2)Alj^ (*Gli1*-CreER^T2^, 007913 Jackson laboratories), B6.129×1-Gt(ROSA)26Sor^tm1(EYFP)Cos^/J (*R26R*.EYFP, 006148 Jackson laboratories), *Gli1*-EGFP^79^ and EYFP mice (obtained by crossing *R26R*.EYFP with the relevant Cre mice) were bred and maintained at the Bio-Facility animal house (Technical University of Denmark). C57BL/6Nrj mice were purchased from Janvier Labs (Le Genest-Saint-Isle, France). *Ackr4*^tmlCcbl1^ mice (*Ackr4*.EGFP)^80^ were bred and maintained in the Central Research Facility, Glasgow University. Mice were maintained on a 12 h light and dark cycle at 22 °C and 55% humidity. Adult mice were used between 5.5 and 12w of age. Mice of both genders were used in all experiments and littermates were used as controls. All experiments were approved by the Danish Animal Experiments Inspectorate (license 2016-15-0201-00931), or with ethical approval under a Project Licence from the UK Home Office.

### Kidney grafting

EYFP male mice were mated overnight with C57BL/6Nrj females and the following morning was defined as gestational day 0.5 (E0.5). Pregnant dams were sacrificed at E12.5 and small and large intestine were dissected from embryos under a stereo microscope (VWR). Adult WT mice were anaesthetized by i.p injection of Ketaminol Vet. (100 mg/kg, MSD animal health) and Rompun Vet. (10 mg/kg, Bayer) and were injected subcutaneously with Bupaq (0.1 mg/kg, Richter Pharma). Washed embryonic intestine was transplanted under the kidney capsule of anesthetized recipients as described previously^42^. Recipients were sacrificed at the time points indicated and grafts were dissected and cut into pieces prior to cell isolation as described below.

### In vivo lineage tracing

*Gli1*-CreER^T2^ male mice were mated overnight with *R26R*.EYFP females and the following morning was defined as gestational day E0.5. At E11.5, pregnant dams were injected i.p. with 4-hydroxytamoxifen ((4-OHT), 1.6 mg, Sigma) and progesterone (0.8 mg, Sigma) in 160 μL phosphate buffered saline (PBS) with 25% Kolliphor (Sigma)/25% ethanol (Fischer Scientific). Small and large intestine were isolated from embryos or weaned offspring at the time points indicated.

### Cell isolation

Intestinal cell suspensions were generated as described previously^81^ with minor changes. Briefly, washed intestinal tissue was opened longitudinally and Peyer’s patches removed. For scRNA-seq and bulk RNA-seq experiments on adult intestine, muscularis externa was stripped away using tweezers. Tissues were cut into 0.5–1 cm pieces and epithelial cells removed by 3 consecutive rounds of incubation in HBSS supplemented with HEPES (15 mM), sodium pyruvate (1 mM), penicillin/streptomycin (100 U/mL), gentamycin (0.05 mg/mL), EDTA (2 mM) (all Invitrogen) and FCS (2.5%) (Sigma), for 15 min at 37 °C with constant shaking at 350 rpm. After each incubation, samples were shaken for 10 sec and medium containing epithelial cells and debris was discarded. For colonic tissues, DL-dithiothreitol (5 mM) (Sigma) was added at the first incubation step. Remaining tissue pieces were digested with collagenase P (0.6 U/mL, Sigma) or with Liberase TM (0.325 U/mL, Roche) and DNAse I (31 μg/mL, Roche) in R10 medium (RPMI 1640, sodium pyruvate (1 mM), HEPES (10 mM), penicillin/streptomycin (100 U/mL), gentamycin (0.05 mg/mL), and 10% FCS) for up to 30 min at 37 °C with constant shaking at 550 rpm (small intestine) or with a magnetic stirrer and at 280 rpm (large intestine). For bulk RNA-seq cells were treated with ACK lysing buffer (Gibco) to lyse red blood cells prior to sorting. For isolation of cells from embryonic intestine, tissues were digested directly for 30 min at 37 °C in Eppendorf tubes with constant shaking at 900 rpm. The resulting cell suspensions were filtered through a 70 μm filter and washed in MACS buffer (PBS with FCS (3%) and EDTA (2 mM)) twice prior to subsequent analyses.

### Flow cytometry and cell sorting

Cell suspensions were stained with fluorochrome labeled primary antibodies (see Supplementary Table 1) in Brilliant stain buffer (BD Biosciences) for 30 min on ice. Flow cytometry was performed on an LSR Fortessa II (BD Biosciences), FACSAria Fusion (BD Biosciences), or FACSMelody (BD Biosciences) and analyzed with FlowJo software (TreeStar). Dead cells were identified by staining with either 7-AAD (eBioscience) or Zombie UV fixable viability dye (BD Biosciences) and cell doublets were excluded on the basis of FSC-A/FSC-H. For intracellular staining, cells were stained for surface antigens, fixed with FoxP3 Staining Buffer set (eBioscience) and stained for αSMA in FoxP3 Permeabilization buffer (eBioscience). After washing, cells were stained with antibodies to surface antigens not compatible with fixation according to the manufacturer’s instructions.

### Immunohistochemistry

Tissues were fixed in paraformaldehyde (4%, PFA) and sectioned (70 μm) using a Vibratome (Leica VT12000S). Sections were incubated in PBS containing bovine serum albumin (BSA) (1%) and Triton-X100 (0.3%) for 1 h at room temperature (RT) to block non-specific staining and incubated with fluorochrome conjugated or unconjugated primary antibodies (see Supplementary Table 1) overnight at 4 °C. After washing with PBS containing Triton-X100 (0.3%), tissues were incubated with secondary antibodies (see Supplementary Table 1) at RT for 2–4 h. For detection of CD81, staining with biotinylated anti-CD81 was enhanced using the Biotinyl Tyramide kit (Perkin Elmer) according to the manufacturer’s instructions after blocking of endogenous biotin using Streptavidin/Biotin Blocking kit (Invitrogen). Endogenous. peroxidase was inactivated by incubating tissues with H_2_O_2_ (3%) for 30 min at RT before incubation with streptavidin-horse radish peroxidase (HRP). Sections were analyzed under 40x magnification using a Zeiss LSM710 confocal microscope and images were processed using Zeiss Zen and Imaris software. For histological analysis of kidney grafts, tissue pieces were fixed in 4% paraformaldehyde for 8 h and paraffin-embedded sections were stained with hematoxylin and eosin.

### RNAscope

Sections (3–5 μm) of formalin-fixed paraffin-embedded tissues were transferred to glass slides and RNA-scope staining was performed using the RNAscope® Multiplex Fluorescent Reagent Kit v2 (ACDBio) according to the manufacturer’s instructions with the indicated probes (see Supplementary Table 1). Tissue sections were incubated with anti-PDPN in RNAscope® co-detection Antibody Diluent overnight at 4 °C, slides were washed in PBS and Tween-20 (0.1%), permeabilized and hybridized with probes. Probes were detected using the Opal^TM^ fluorochromes, anti-PDPN was detected by incubation with Cy3 conjugated anti-syrian hamster antibody for 30 min at RT, and nuclei were counter-stained using DAPI. Stained samples were washed in H_2_O and a cover glass with ProLong Gold antifade mount applied. Samples were imaged with a Zeiss LSM 710 confocal laser microscope.

### Library preparation and sequencing

#### Single-cell RNA-seq

Sorted cells were washed in cold PBS containing bovine serum albumin (0.04%), counted and diluted to the desired concentration following 10X Genomics guidelines (10x Genomics, CG000053_CellPrepGuide_RevC). ScRNA-seq libraries were prepared according to the manufacturer’s instructions using Chromium Single Cell 3′ Library & Gel Bead Kit v3 (10x Genomics, PN-1000092) or 5′ kit Chromium Single Cell 5′ Library & Gel Bead Kit (10x Genomics, PN-1000006) and Chromium Chip B Single Cell Kit (PN-1000074) with the Chromium Controller & Next GEM Accessory Kit (10x Genomics, PN-120223). In brief, single cells, reverse transcription reagents, Gel Beads containing barcoded oligonucleotides, and oil were combined on a microfluidic chip to form Gel Beads in Emulsion (GEMs). Individual cells were lysed inside the GEMs and the released poly-A transcripts were barcoded with an Illumina R1 sequence, a 10X barcode and a Unique Molecular Identifier (UMI) during reverse transcription (RT). After RT, GEMs were broken, barcoded cDNA was purified using Dynabeads MyOne silane (10x Genomics, PN-2000048) and amplified by Polymerase Chain Reaction (PCR). Amplified cDNA were cleaned up with SPRIselect Reagent kit (Beckman Coulter, B23318). Indexed sequencing libraries were constructed by enzymatic fragmentation, end-repair and A-tailing, before a second and final PCR amplification using the Chromium i7 Sample Index (10x Genomics, PN-220103), introducing an Illumina R2 sequence, a unique sample index (allowing multiplex sequencing) and P5/P7 Illumina sequencing adapters to each library. Library quality control and quantification were performed using a KAPA Library Quantification Kit for Illumina Platforms (Kapa Biosystems, KK4873) and the 2100 Bioanalyzer equipped with a High Sensitivity DNA kit (Agilent, 5067-4626). Muliplexed libraries were pooled and sequenced either by NextSeq 500/550 High Output v2.5 kit (150 cycles) at the Center of Excellence for Fluorescent Bioanalytics (KFB, University of Regensburg, Germany) or by Novaseq 6000 S1 or S2 (200 cycles) at the SNP&SEQ Technology Platform (Uppsala, Sweden) with the following sequencing run parameters: Read1 − 28 cyles; i7 index − 8 cycles; Read2 – 126 cycles at a depth of at least 100 M reads/sample.

#### Bulk RNA-seq

MSC subsets were sorted into RLT buffer and total RNA was isolated using the RNeasy Micro kit (Qiagen). Following the manufacturer’s protocol, extraction was performed with an on-column DNAse digestion step after the first washing step. The RNA quality and quantity were measured using the 2100 BioAnalyzer equipped with RNA6000 Pico chip (Agilent Technologies). Using Ovation RNA-Seq System V2 kit (Nugen), RNA was subjected to whole transcriptome amplification and the MiniElute Reaction Cleanup kit (Qiagen) was used to purify the amplified cDNA samples. The quantity and quality of the cDNA samples were measured using the 2100 BioAnalyzer equipped with DNA1000 chip (Agilent technologies) and the Nanodrop (ThermoFisher Scientific). Following the manufacturer’s instructions, libraries were constructed with the Ovation Ultralow system V2 kit (Nugen). A Bioruptor Pico (Diagenode) was used to fragment amplified cDNA (100 ng) by sonication, and sheared cDNA end-repaired to generate blunt ends and ligated to Illumina adapters with indexing tags followed by AMPure XP bead purification. A 2100 Bioanalyzer equipped with DNA1000 chip (Agilent technologies) was used to evaluate library size distribution, and this was quantified using KAPA library Quantification Kit Illumina platforms (Kapa Biosystems). Libraries were diluted before being pooled at equimolar concentration (10 nM final) and subsequently sequenced on the Hiseq2500 platform (Illumina) using 50 bp single reads (Center for Genomic Regulation, Spain) with a read depth of 15–20 M reads per sample.

### Computational analysis

#### Single-cell RNA-seq

Alignment of scRNA-seq data to mouse reference genome, mm10, was performed with CellRanger (version 3.0.2 & 3.1.0)^82,83^. The data was imported into R (version 4.0.1)^84^ and processed to remove debris and doublets in individual samples by looking at gene, read counts and mitochondrial gene expression. Variable genes were calculated with Seurats FindVariableFeatures function and selection method set to “vst” (Seurat version 3.1.5)^85^. The respective samples and all their overlapping genes were then integrated with anchor integration for Seurat. Cell cycle effects were regressed out with linear regression using a combination of the build-in function in Seurat and scoring gene sets from ccremover (version 1.0.4) per cell^86^ during scaling of the gene expression. The datasets were dimensionality reduced first with PCA and then UMAP and clustered with Louvain clustering all using Seurat. After initial clustering, contaminating cells were removed and an additional round of clustering and dimensionality reduction with UMAP was run on the cells of interest. DEGs were identified using Seurat FindAllMarkers function with the default test setting (non-parameteric Wilcoxon Rank Sum test. correcting for multiple comparisons with Bonferroni). Expression of gene modules in the form of published signature gene sets were calculated with AddModuleScore (Seurat) taking the top DEG from telocytes (10 genes), Lo-1 FB (10 genes) and Lo-2 FB (7 genes) reported by McCarthy et al.^7^, the top 10 DEG from FB1-5, MC and SMCs reported by Hong et al.^11^, and the top 20 DEG from *Pi16*^*+*^, *Col15a1*^*+*^, *Fbln1*^*+*^ and *Bmp4*^*+*^ FB reported by Buechler et al.^60^. Pearson correlations between datasets were calculated based on average expressions per cluster of overlapping variable genes and plotted with heatmap.2 (version 3.0.3)^87^. Single-cell heatmaps were constructed with a modified version of Seurats DoHeatmap to allow for multiple grouping variables. Data plotted in expression heatmaps was scaled based on the anchor integrated data. Data imputation was performed per dataset across samples on raw count data with magicBatch^58^ (version 0.1.0) where the affinity matrix used was Seurat’s batch-corrected PCA coordinates with *T* = 6. Trajectories and trajectory spaces were determined with tSPACE^57^ (version 0.1.0) on the top 2000 imputed variable genes for adult trajectories and top 1000 imputed variable genes for the integrated E12.5 and adult trajectory. The tPC1–3 3D trajectory spaces were embedded into 2D using Dufy^88^ (version 1.0.1). Splicing information was calculated from output BAM files with velocyto^61^ (version 0.17.17), with a repeated annotation file^89^. In R with velocyto.R (version 0.6), genes were filtered using a threshold of 0.2 for spliced and 0.05 for un-spliced matrices. The remaining variable genes were used to estimate the relative genes velocities for delta*T* = 1 on the cells of interest with a quantile fit of 0.02 and kCells = 40. The velocity estimates were then embedded on the tPC space with *n* = 400 and scale = sqrt.

#### Bulk RNA-seq

Raw RNA sequencing data from the 30 samples were pre-processed with TrimGalore (version 0.4.0) and FastQC (version 0.11.2). Pseudo-alignment of reads was performed with Kallisto (version 0.42.5) to obtain RNA expression information. To assess correlations between bulk-seq samples and SC clusters Pearson correlations based on SC variable genes were calculated between the bulk-seq samples and the pseudo-bulk of the SC clusters for the individual tissues and visualized with heatmap.2 (part of gplot package).

For all DESeq2 (1.26.0^90^) analysis, transcripts identified in less than 3 replicates and at levels below 6 reads were filtered out prior to further analysis. Heatmaps of bulk-seq data expression was created in R with the ComplexHeatmap package (version 2.7.11) and volcano plots with ggplot2 (version 3.3.1). For the comparison between tissues, DEGs were only classified as significant if they had a |log_2_FC| > 1.5 and adjusted (Benjamini-Hochberg corrected) *p* value <0.05. GO analysis was performed using GO Biological Processes 2021^35,36^ from Enrichr computational biosystems^91–93^ and plotted with ggradar (version 0.2) using sqrt(−log10(adjusted *p* values)) transformation.

### Statistical analysis

Statistical significance was determined with a two-way ANOVA with Benjamini, Krieger and Yekutieli multiple comparisons and performed in Prism software (GraphPad). *p < 0.05, **p < 0.01, ***p < 0.001.

### Reporting summary

Further information on research design is available in the Nature Portfolio Reporting Summary linked to this article.

## Supplementary information

Supplementary Information

Description of additional supplementary files

Supplementary Data 1

Supplementary Data 2

Supplementary Data 3

Reporting Summary

## Data Availability

The single-cell RNA-seq and bulk RNA-seq data has been deposited at NCBI GEO under the accession code “GSE182176”. The flow cytometry-based data generated in this study are provided in the Source Data file associated with this manuscript. All other relevant data supporting the key findings of this study are available within the article and its Supplementary Information files or from the corresponding author upon reasonable request. Source data are provided with this paper.

## Source data

Source Data
