# Supplementary material for: The small and large intestine contain related mesenchymal subsets that derive from embryonic Gli1+ precursors
Source: Nat Commun. 2023 Apr 21;14:2307. doi: 10.1038/s41467-023-37952-5 (PMC10121680; doi:10.1038/s41467-023-37952-5)
Supplement: Supplementary file 1 — Supplementary Information [file 41467_2023_37952_MOESM1_ESM.pdf]

Supplemental Information

The small and large intestine contain transcriptionally related mesenchymal stromal cell subsets that derive from embryonic *Gli1*$^+$ precursors

Simone Isling Pærregaard[1], Line Wulff[1, #], Sophie Schussek[1, #], Kristoffer Niss[2], Urs Mörbe[1], Johan Jendholm[1], Kerstin Wendland[3], Anna T. Andrusaite[4], Kevin F. Brulois[5], Robert J. B. Nibbs[4], Katarzyna Sitnik[1], Allan McI Mowat[4], Eugene C. Butcher[5,6], Søren Brunak[2], William W. Agace[1,3, *].

Supplementary Table 1: Antibodies and RNA-scope probes

Antibodies			Dilution
AF488 donkey anti-rat IgG	Jackson IR	Cat#712-545-153, RRID: AB_2340684	1mg/mL, 1/500
AF488 anti-mouse αSMA clone 1A4	Abcam	Cat#ab184675, RRID: AB_2832195	1/200-1/300
AF488 streptavidin	Thermo Fischer Scientific (TFS)	Cat#S32354, RRID: AB_2315383	1/500
AF594 anti-mouse CD31 clone MEC13.3	BioLegend	Cat#102520, RRID: AB_2563319	1/100-1/150
AF647 anti-mouse CD31 clone MEC13.3	BioLegend	Cat#102515, RRID: AB_2161030	1/75-1/200
AF647 donkey anti-goat IgG	Jackson IR	Cat#705-605-147, RRID: AB_2340437	1mg/mL, 1/500
AF647 donkey anti-rabbit IgG	Jackson IR	Cat#711-605-152, RRID: AB_2492288	1mg/mL, 1/500
AF647 anti-mouse EpCAM clone G8.8	BioLegend	Cat#118211, RRID: AB_1134104	1/200
AF700 anti-mouse B220 clone RA3-6B2	BioLegend	Cat#103232; RRID: AB_493717	1/100
AF700 anti-mouse CD11b clone M1/70	BioLegend	Cat#101222, RRID: AB_493705	1/100
AF700 anti-mouse CD11c clone N418	BioLegend	Cat#117320, RRID: AB_528736	1/100
AF700 anti-mouse CD45 clone 30-F11	TFS	Cat#56-0451-82, RRID: AB_891454	1/100
AF700 anti-mouse CD45.2 clone 104	TFS	Cat#56-0454-82, RRID: AB_657752	1/100
AF700 anti-mouse Gr-1 clone RB6-8C5	BioLegend	Cat#108422, RRID: AB_2137487	1/100
AF700 anti-mouse NK1.1 clone PK136	BioLegend	Cat#108730, RRID: AB_2291262	1/100
AF700 anti-mouse Ter119 clone Ter119	BioLegend	Cat#116220, RRID: AB_528963	1/100
APC anti-mouse BP3 clone BP3	BioLegend	Cat#140208, RRID: AB_10901172	1/250
APC anti-mouse L1CAM clone 555	Miltenyi	Cat#130-102-221, RRID: AB_2655594	1/40-1/100
APC anti-mouse NCAM clone 809220	R&D	Cat#FAB7820A	1/100
APCCy7 anti-mouse CD90.2 clone 53-2.1	BD Biosciences	Cat#561641, RRID: AB_10898013	1/100
APCCy7 anti-mouse CD45.2 clone 104	BioLegend	Cat#109824, RRID: AB_830789	1/100
APCCy7 anti-mouse EpCAM clone G8.8	BioLegend	Cat#118218, RRID: AB_2098648	1/250

APC-eF780 anti-mouse Ter119 clone Ter119	TFS	Cat#47-5921-82, RRID: AB_1548786	1/100
Biotin anti-mouse CD34 clone MEC14.7	BioLegend	Cat#119304; RRID: AB_345282	1/100
Biotin anti-mouse CD81 clone Eat-2	BioLegend	Cat#104903, RRID: AB_313138	1/100
BUV395 anti-mouse CD146 clone ME-9F1	BD Biosciences	Cat#740330, RRID: AB_2740063	1/100
BV421 anti-mouse PDGFRα clone APA5	BD Biosciences	Cat#566293, RRID: AB_2739666	1/66
BV421 anti-mouse CD34 clone RAM34	BD Biosciences	Cat#562608, RRID: AB_11154576	1/100
BV510 anti-mouse EpCAM clone G8.8	BD Biosciences	Cat#747748, RRID: AB_2872217	1/100-1/250
BV510 streptavidin	BD Biosciences	Cat#563261, RRID: AB_2869477	1/100
BV605 anti-mouse CD31 clone 390	BioLegend	Cat#102427, RRID: AB_2563982	1/250
BV605 anti-mouse Itgb1 clone HM β1-1	BD Biosciences	Cat#740365, RRID: AB_2740097	1/200
BV650 anti-mouse BP3 clone BP-3	BD Biosciences	Cat#740611, RRID: AB_2740311	1/100
BV650 anti-mouse CD31 clone 390	BD Biosciences	Cat#740483, RRID: AB_2740207	1/250-1/400
BV711 anti-mouse CD9 clone KMC8	BD Biosciences	Cat#740696, RRID: AB_2740380	1/100
BV786 anti-mouse BP3 clone BP-3	BD Biosciences	Cat#741012, RRID: AB_2740634	1/100-1/200
Cy3 donkey anti-rat IgG	Jackson IR	Cat#712-166-150, RRID: AB_2340668	1.5mg/mL, 1/500
Cy3 goat anti Syrian hamster IgG	Jackson IR	Cat#107-165-142, RRID: AB_2337464	2mg/mL, 1/500
FITC anti-mouse CD34 clone RAM34	TFS	Cat#11-0341-85, RRID: AB_465022	1/100-1/150
FITC anti-mouse CD90.2 clone 53-2.1	TFS	Cat#11-0902-82, RRID: AB_465154	1/100
PE anti-mouse CD141 clone REA964	Miltenyi	Cat#130-116-017, RRID: AB_2727308	1/100
PE anti-mouse CD26 clone H194-112	BioLegend	Cat#137804, RRID: AB_2293047	1/100
PE anti-mouse ESAM clone 1G8	BioLegend	Cat#136204, RRID: AB_1953301	1/200
PE anti-mouse Itgb1 clone HMb1-1	BioLegend	Cat#102208, RRID: AB_312885	1/200
PE/CF594 anti-mouse PDGFRα clone APA5	BD Biosciences	Cat#562775, RRID: AB_2737786	1/66-/100
PECy7 anti-mouse PDPN clone 8.1.1	TFS	Cat#25-5381-82, RRID: AB_2573460	1/125-1/500
PerCP/Cy5.5 anti-mouse CD31 clone 390	BioLegend	Cat#102420, RRID: AB_10613644	1/200
PerCP-eF710 anti-mouse EpCAM clone G8.8	TFS	Cat#46-5791-82, RRID: AB_10598205	1/250
Unconjugated anti-mouse CD34 clone RAM34	TFS	Cat#14-0341-82, RRID: AB_467210	1/150-1/200
Unconjugated anti mouse PDPN clone 8.1.1	Biolegend	Cat#127402, RRID: AB_1089187	1/250
Unconjugated anti-mouse PPARγ polyclonal	Invitrogen	Cat#PA5-25757, RRID: AB_2543257	1/100
Unconjugated anti-mouse PDGFRα polyclonal	R&D	Cat#AF1062, RRID: AB_2236897	1/300-1/400
**RNA-scope probes**			
RNAscope® Probe - Mm- CXCL14-C3	ACDBio	459741-C3	
RNAscope® Probe- Mm-Fgfr2-no-XHs-C1	ACDBio	443501	

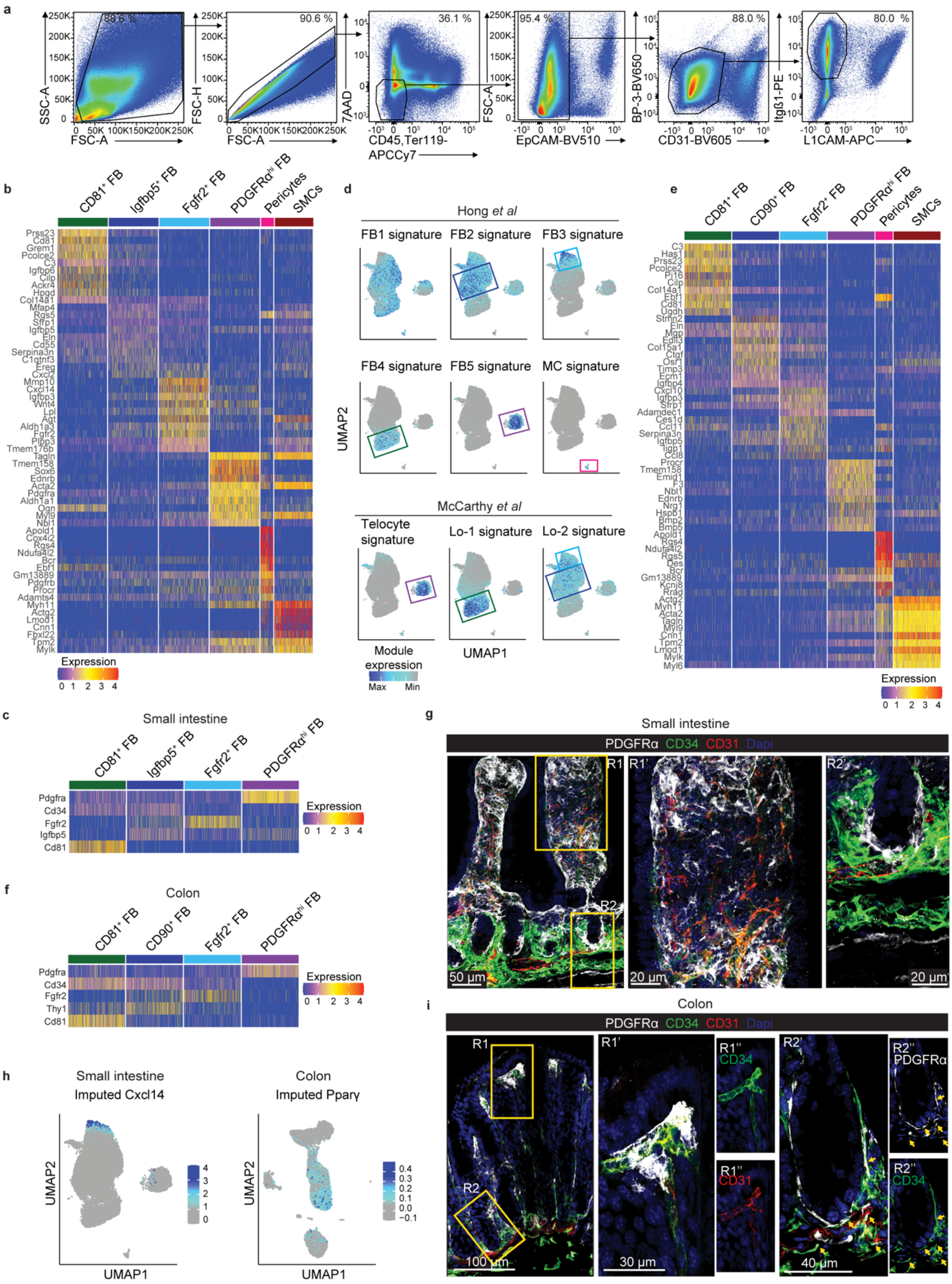
a
SSC-A
FSC-A
88.6 %
FSC-H
90.6 %
7AAD
CD45,Ter119-APCCy7
36.1 %
FSC-A
EpCAM-BV510
95.4 %
BP-3-BV650
CD31-BV605
88.0 %
Itgβ1-PE
L1CAM-APC
80.0 %
b
CD81+ FB
Igfbp5+ FB
Fgfr2+ FB
PDGFRαhi FB
Pericytes
SMCs
Prss23
Cd81
Grem1
Pcolce2
C3
Igfbp6
Cilp
Ackr4
Hpgd
Col14a1
Mfap4
Rgs5
Sfrp1
Igfbp5
Eln
Cd55
Serpina3n
C1qtnf3
Ereg
Cxcl2
Mmp10
Cxcl14
Igfbp3
Wnt4
Lpl
Agt
Aldh1a3
Fgfr2
Plpp3
Tmem176b
Tagln
Tmem158
Sox6
Ednrb
Acta2
Pdgfra
Aldh1a1
Ogn
Myl9
Nbl1
Apold1
Cox4i2
Rgs4
Ndufa4l2
Bcr
Ebf1
Gm13889
Pdgfrb
Procr
Adamts4
Myh11
Actg2
Lmod1
Cnn1
Fbxl22
Tpm2
Mylk
Expression
0 1 2 3 4
c
Small intestine
CD81+ FB
Igfbp5+ FB
Fgfr2+ FB
PDGFRαhi FB
Pdgfra
Cd34
Fgfr2
Igfbp5
Cd81
Expression
0 1 2 3 4
d
Hong et al
FB1 signature
FB2 signature
FB3 signature
FB4 signature
FB5 signature
MC signature
UMAP2
McCarthy et al
Telocyte signature
Lo-1 signature
Lo-2 signature
Module expression
Max
Min
UMAP1
e
CD81+ FB
CD90+ FB
Fgfr2+ FB
PDGFRαhi FB
Pericytes
SMCs
Expression
0 1 2 3 4
f
Colon
CD81+ FB
CD90+ FB
Fgfr2+ FB
PDGFRαhi FB
Pdgfra
Cd34
Fgfr2
Thy1
Cd81
Expression
0 1 2 3 4
g
Small intestine
PDGFRα CD34 CD31 Dapi
R1
R1'
R2
R2'
50 µm
20 µm
20 µm
h
Small intestine
Imputed Cxcl14
UMAP2
UMAP1
Colon
Imputed Pparγ
i
Colon
PDGFRα CD34 CD31 Dapi
R1
R1'
R1''
CD34
R1''
CD31
R2
R2'
R2''
PDGFRα
R2''
CD34
100 µm
30 µm
40 µm

Figure S1.

Related to Figure 1. (**a**) Flow cytometric gating strategy for sorting adult small intestinal and colonic Itgβ1^{+} MSC. (**b**) Heatmap with transcription levels (integrated data) of the top 10 differentially expressed genes (DEG) between adult small intestinal MSC subsets. (**c**) Heatmap with transcription levels (anchor integrated data) of markers used to distinguish small intestinal FB subsets. (**d**) Signature genes for MSC subsets identified in Hong *et al*[1] and McCarthy *et al*[2] projected onto the small intestinal MSC UMAP as gene modules. Boxes represent the 6 MSC clusters and are color coded as in (**b**). (**e**) Heatmap with transcription levels (integrated data) of the top 10 DEGs between adult colonic MSC subsets. (**f**) Heatmap with transcription levels (anchor integrated data) of markers used to distinguish colonic FB subsets. (**g** and **i**) Immunohistochemical staining of mouse jejunum (**g**) or colon (**i**) for indicated antigens. R1' and R2' are high magnifications of the R1 and R2 quadrants (yellow squares) in the left image. (**i**) Arrows indicate CD34^{+} FB (CD34^{+}PDGFRα^{+}CD31^{-} cells). Results are representative stains from (**g**) 3 and (**i**) 2 experiments analyzing intestinal sections from 3 mice/experiment. (**h**) MAGIC imputed projection of *Cxcl14* on small intestinal MSC UMAP (left) and *Pparγ* expression projected onto the colon MSC UMAP (right).

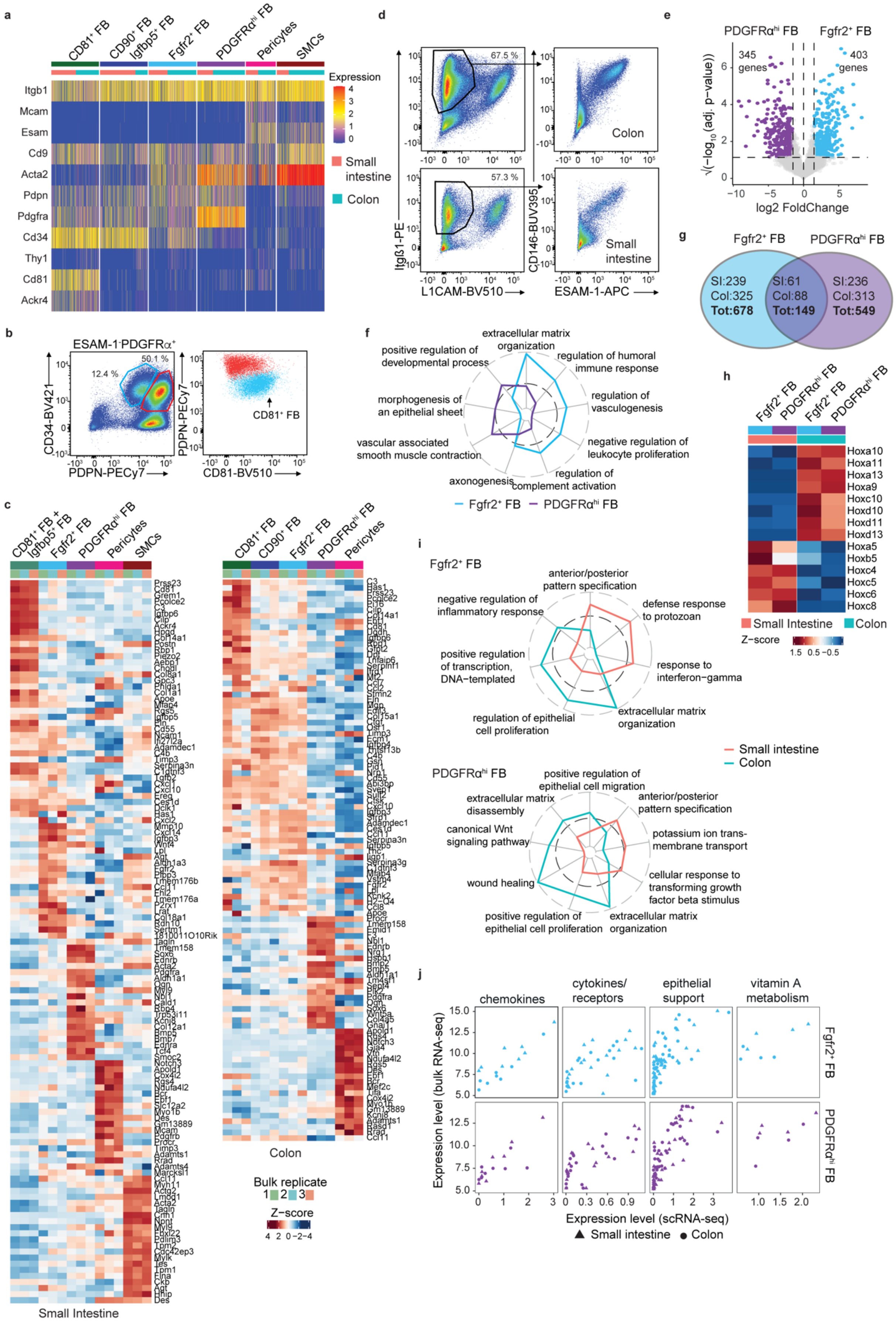

a
CD81⁺ FB
CD90⁺ FB
Igfbp5⁺ FB
Fgfr2⁺ FB
PDGFRα^hi FB
Pericytes
SMCs
Itgb1
Mcam
Esam
Cd9
Acta2
Pdpn
Pdgfra
Cd34
Thy1
Cd81
Ackr4
Expression
Small intestine
Colon
b
ESAM-1⁻PDGFRα⁺
12.4 %
50.1 %
CD34-BV421
PDPN-PECy7
CD81⁺ FB
CD81-BV510
c
Small Intestine
Colon
Bulk replicate
1 2 3
Z−score
d
67.5 %
57.3 %
Itgß1-PE
L1CAM-BV510
CD146-BUV395
ESAM-1-APC
Colon
Small intestine
e
PDGFRα^hi FB
Fgfr2⁺ FB
345 genes
403 genes
√(−log10 (adj. p−value))
log2 FoldChange
f
extracellular matrix organization
regulation of humoral immune response
regulation of vasculogenesis
negative regulation of leukocyte proliferation
regulation of complement activation
axonogenesis
vascular associated smooth muscle contraction
morphogenesis of an epithelial sheet
positive regulation of developmental process
Fgfr2⁺ FB
PDGFRα^hi FB
g
Fgfr2⁺ FB
PDGFRα^hi FB
SI:239 Col:325 Tot:678
SI:61 Col:88 Tot:149
SI:236 Col:313 Tot:549
h
Fgfr2⁺ FB
PDGFRα^hi FB
Hoxa10
Hoxa11
Hoxa13
Hoxa9
Hoxc10
Hoxd10
Hoxd11
Hoxd13
Hoxa5
Hoxb5
Hoxc4
Hoxc5
Hoxc6
Hoxc8
Small Intestine
Colon
Z-score
i
Fgfr2⁺ FB
anterior/posterior pattern specification
defense response to protozoan
response to interferon−gamma
extracellular matrix organization
regulation of epithelial cell proliferation
positive regulation of transcription, DNA−templated
negative regulation of inflammatory response
PDGFRα^hi FB
positive regulation of epithelial cell migration
anterior/posterior pattern specification
potassium ion trans-membrane transport
cellular response to transforming growth factor beta stimulus
extracellular matrix organization
positive regulation of epithelial cell proliferation
wound healing
canonical Wnt signaling pathway
extracellular matrix disassembly
Small intestine
Colon
j
chemokines
cytokines/ receptors
epithelial support
vitamin A metabolism
Expression level (bulk RNA-seq)
Expression level (scRNA-seq)
Fgfr2⁺ FB
PDGFRα^hi FB
Small intestine
Colon

Figure S2.

Related to Figure 2. (**a**) Heatmap showing transcription levels (integrated data) of genes used as markers to distinguish the 6 MSC subsets by flow cytometry. (**b**) Flow cytometric analysis verifying that colonic $PDPN^{lo}CD34^{+}$FB (blue gate, left plot) express CD81 (blue cells right plot). Representative gating of at least 3 experiments with 4-7 mice/experiment. (**c**) Heatmaps showing scaled transcription levels of the top 20 DEGs defining the scRNA-seq subsets within the bulk RNA-seq subsets from colon (left) and small intestine (right). (**d**) Flow cytometric gating strategy of colon (top panels) and small intestine (bottom panels) showing co-staining of ESAM-1 and CD146 by $Itg\beta1^{+}$ MSCs. Representative stains of at least 6 experiments with 1 mouse/experiment. (**e**) Volcano plot displaying DEGs between $PDGFR\alpha^{hi}$ and $Fgfr2^{+}$ FB irrespective of location ($Fgfr2^{+}$ FB n=6, $PDGFR\alpha^{hi}$ FB n=6), adjusted p-values by Benjamini-Hochberg correction. (**f**) Radarplot of sqrt(-log10(p-adj)) of gene ontology (GO) analysis using Enrichr (GO Biological Processes 2021) of DEGs between in $PDGFR\alpha^{hi}$ or $Fgfr2^{+}$ FB. Black dashed line indicates the threshold of adjusted p-value=0.05. Selected significant GO terms are shown. (**g**) Venn diagram depicting the number of DEGs in indicated FB subsets between the small intestine (SI) and colon (Col). (**h**) Heatmap of scaled transcription levels (averaged between bulk RNA-seq triplicates) of Hox genes that were differentially expressed between small intestine and colon in the indicated FB subset. (**i**) Radarplot of sqrt(-log10(p-adj)) for GO analysis of DEGs between small intestinal and colonic $PDGFR\alpha^{hi}$ FB and between small intestinal and colonic $Fgfr2^{+}$ FB. Significant terms of biological relevance are included in this visualization. (**j**) Gene expression values between bulk RNA-seq and scRNA-seq data for the gene sets analyzed in Figure 2h-j.

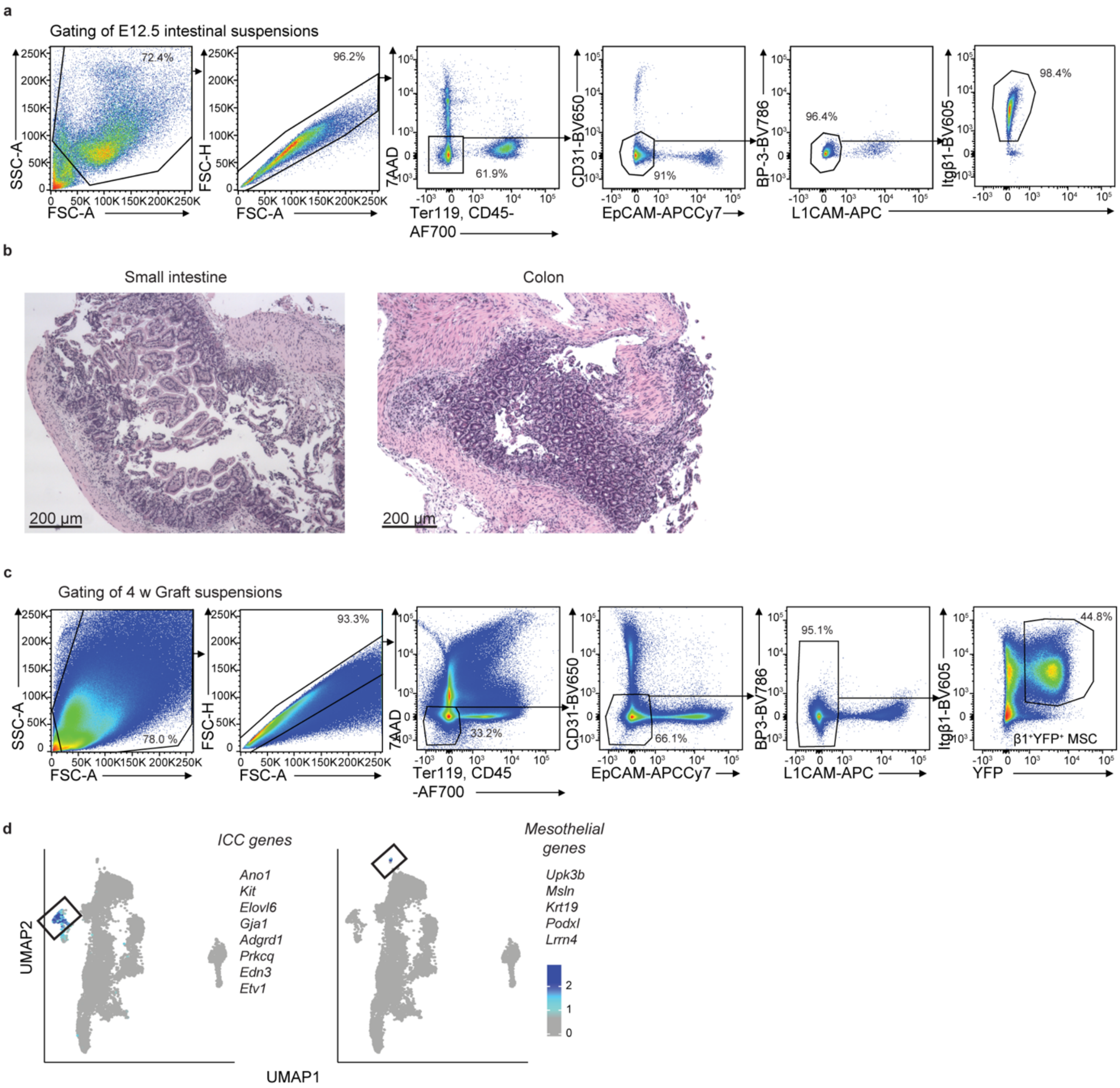

Figure S3.

Related to Figure 3. (**a**) Flow cytometric gating strategy for the identification and sorting of intestinal Itgβ1^{+} MSC from embryonic day (E)12.5 mice. (**b**) Hematoxylin and eosin staining of small intestinal and colonic grafts 6 weeks post transplantation. Results are representative stains of 2 grafts for each tissue. (**c**) Flow cytometric gating strategy for the identification and sorting of YFP^{+}Itgβ1^{+} MSC from intestinal grafts. (**d**) Projections of module score of signature genes for Interstitial cells of Cajal (ICC) and mesothelium onto colon graft UMAP. Boxes identify the ICC and mesothelial clusters.

Supplementary Figure 4

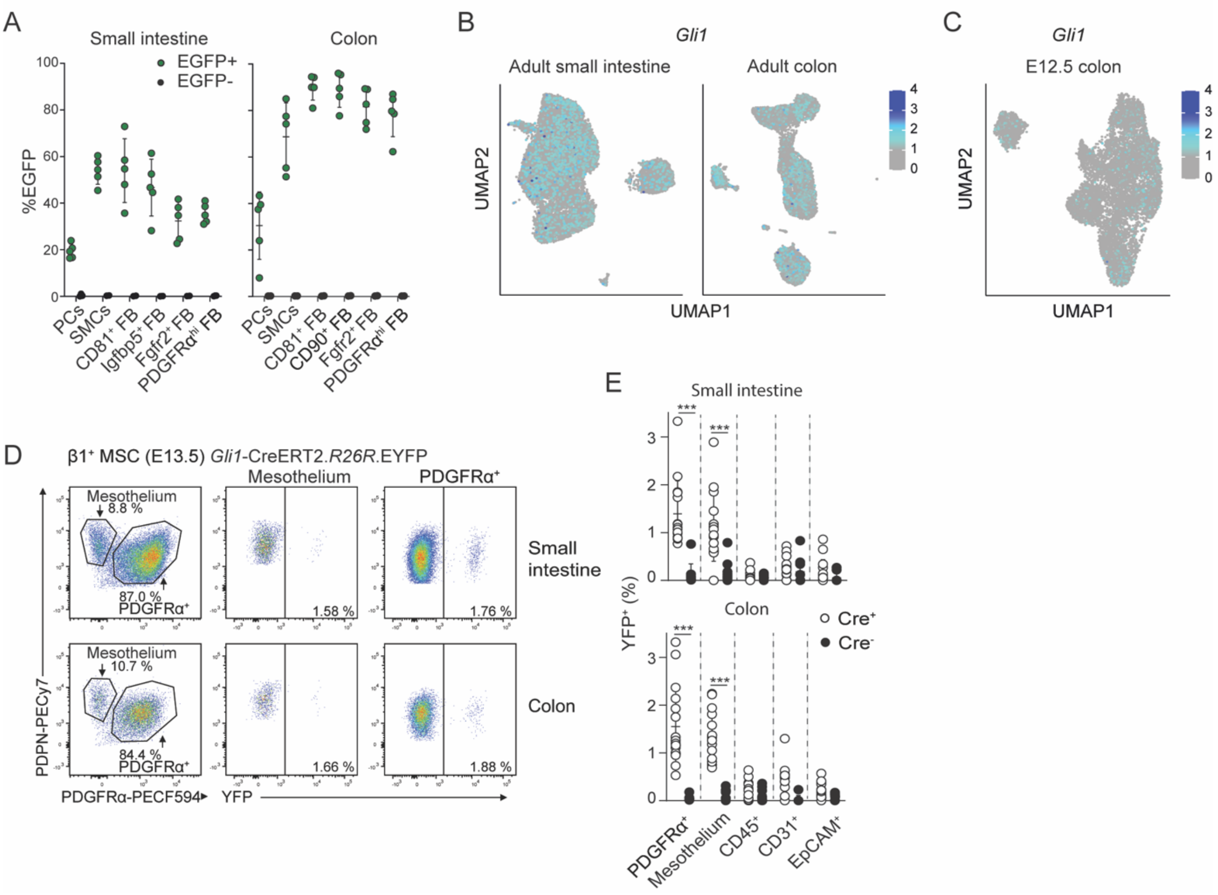

Figure S4.

Related to Figure 4. (**a**) Proportions of EGFP^{+} cells within indicated MSC subsets from the small intestine (left panel) and colon (right) of 7-8 week old *Gli1*-EGFP mice as assessed by flow cytometry. Results are from 8 mice, with each symbol representing an individual mouse and bars show mean and SD. (**b**) *Gli1* log-normalized gene expression projected onto UMAP of adult small intestine (left panel) and colon (right panel) MSC. (**c**) *Gli1* log-normalized gene expression projected onto UMAP of E12.5 large intestine MSC. (**d**) Representative flow cytometric analysis and (**e**) pooled data of the proportions of YFP-expressing cells in indicated intestinal populations of E13.5 *Gli1*.CreERT2$^{+/-}$.*R26R*.EYFP mice 2 days after injection with

4-OHT. Pre-gating strategy as in Fig. S3a. Results are from 4 pooled experiments with 1-6 embryos/experiment (SI, n=25; LI, n=24). Bars, mean (SD). ***$p<0.00001$, 2-way ANOVA with Benjamini, Krieger and Yekutieli multiple comparisons. Source data are provided as a Source Data file.

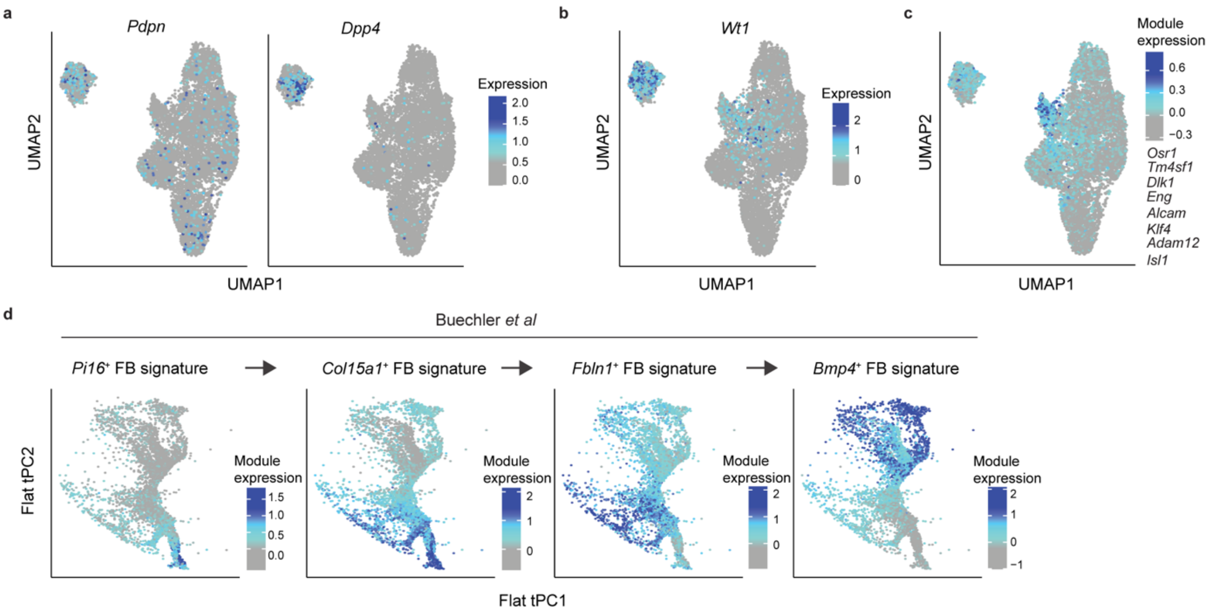

Figure S5.

Related to Figure 5. (**a**) *Pdpn* and *Dpp4*, (**b**) *Wt1* log-normalized gene expressions and (**c**) mesenchymal precursor associated gene expression module score integrated and normalized gene expression projected onto UMAP of E12.5 large intestinal MSC. (**d**) Top 20 DEGs for indicated FB clusters identified in Buechler *et al* projected as gene modules onto colonic tSPACE projections of adult colonic MSC in tPC1-3. Arrows indicate predicted trajectory between FB clusters according to Buechler *et al*[3].

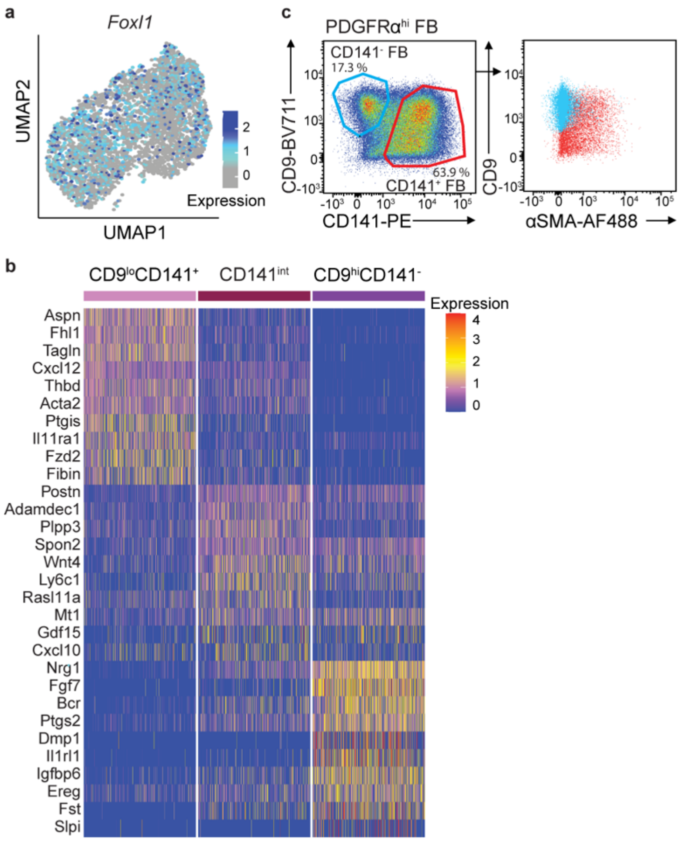

Figure S6.

Related to Figure 6. (**a**) Integrated and normalized *Foxl1* expression projected onto UMAP of colonic PDGFRα^{hi} FB. (**b**) Heatmap with expression (integrated) of top 10 differentially expressed genes (DEGs) between the three colonic PDGFRα^{hi} subsets. (**c**) Flow cytometric analysis of PDGFRα^{hi} FB showing expression of αSMA (right hand plot) by CD141^{+} (red) and CD141^{-} (blue) cells using the gates depicted in the left panel. Results are representative staining of 2 experiments with 3 mice/experiment.

Supplemental references

1. Hong, S. P. *et al.* Distinct fibroblast subsets regulate lacteal integrity through YAP/TAZ-induced VEGF-C in intestinal villi. *Nat. Commun.* **11**, 4102 (2020).
2. McCarthy, N. *et al.* Distinct Mesenchymal Cell Populations Generate the Essential Intestinal BMP Signaling Gradient. *Cell Stem Cell* **26**, 391-402.e5 (2020).
3. Buechler, M. B. *et al.* Cross-tissue organization of the fibroblast lineage. *Nature* (2021). doi:10.1038/s41586-021-03549-5
