# Supplementary material for: The small and large intestine contain related mesenchymal subsets that derive from embryonic Gli1+ precursors
Source: Nat Commun. 2023 Apr 21;14:2307. doi: 10.1038/s41467-023-37952-5 (PMC10121680; doi:10.1038/s41467-023-37952-5)
Supplement: Supplementary file 2 — Description of additional supplementary files [file 41467_2023_37952_MOESM2_ESM.pdf]

Description of additional supplementary files

Supplementary Data File 1: List of differentially expressed genes between intestinal Fgfr2+ and PDGFRαhi fibroblasts.

Supplementary Data File 2: List of differentially expressed genes between small intestinal Fgfr2+ and colonic Fgfr2+ fibroblasts and between small intestinal PDGFRαhi and colonic PDGFRαhi fibroblasts.

Supplementary Data File 3: List of genes that were differentially expressed in both Fgfr2+ and PDGFRαhi fibroblasts in the small intestine versus the colon.
