# Supplementary material for: The small and large intestine contain related mesenchymal subsets that derive from embryonic Gli1+ precursors
Source: Nat Commun. 2023 Apr 21;14:2307. doi: 10.1038/s41467-023-37952-5 (PMC10121680; doi:10.1038/s41467-023-37952-5)
Supplement: Supplementary file 6 — Reporting Summary [file 41467_2023_37952_MOESM6_ESM.pdf]

nature portfolio

Corresponding author(s): William Agace

Last updated by author(s): Mar 24, 2023

Reporting Summary

Nature Portfolio wishes to improve the reproducibility of the work that we publish. This form provides structure for consistency and transparency in reporting. For further information on Nature Portfolio policies, see our Editorial Policies and the Editorial Policy Checklist.

Statistics

For all statistical analyses, confirm that the following items are present in the figure legend, table legend, main text, or Methods section.

n/a	Confirmed	
☐	☒	The exact sample size (*n*) for each experimental group/condition, given as a discrete number and unit of measurement
☐	☒	A statement on whether measurements were taken from distinct samples or whether the same sample was measured repeatedly
☐	☒	The statistical test(s) used AND whether they are one- or two-sided<br>*Only common tests should be described solely by name; describe more complex techniques in the Methods section.*
☒	☐	A description of all covariates tested
☐	☒	A description of any assumptions or corrections, such as tests of normality and adjustment for multiple comparisons
☐	☒	A full description of the statistical parameters including central tendency (e.g. means) or other basic estimates (e.g. regression coefficient) AND variation (e.g. standard deviation) or associated estimates of uncertainty (e.g. confidence intervals)
☐	☒	For null hypothesis testing, the test statistic (e.g. *F*, *t*, *r*) with confidence intervals, effect sizes, degrees of freedom and *P* value noted<br>*Give P values as exact values whenever suitable.*
☒	☐	For Bayesian analysis, information on the choice of priors and Markov chain Monte Carlo settings
☒	☐	For hierarchical and complex designs, identification of the appropriate level for tests and full reporting of outcomes
☐	☒	Estimates of effect sizes (e.g. Cohen's *d*, Pearson's *r*), indicating how they were calculated

Our web collection on statistics for biologists contains articles on many of the points above.

Software and code

Policy information about availability of computer code

Data collection	No data was collected externally, thus no software was used.
Data analysis	ellRanger (version 3.0.2 & 3.1.0), R (version 4.0.1), Seurat (version 3.1.5), ccremover (version 1.0.4), heatmap.2 (version 3.0.3), magicBatch (version 0.1.0), tSPACE (version 0.1.0), Dufy (version 1.0.1), Enrichr (version from october 2022), TrimGalore (version 0.4.0), FastQC (version 0.11.2), Kallisto (version 0.42.5), DESeq2 (version 1.26.0), ggplot2 (version 3.3.1), velocyto.R (version 0.6), velocyto (version 0.17.17), ComplexHeatmap (version 2.7.11), ggradar (version 0.2), FlowJo software (version 10.6), Prism (GraphPad)(version XX)

For manuscripts utilizing custom algorithms or software that are central to the research but not yet described in published literature, software must be made available to editors and reviewers. We strongly encourage code deposition in a community repository (e.g. GitHub). See the Nature Portfolio guidelines for submitting code & software for further information.

Data

Policy information about availability of data

All manuscripts must include a data availability statement. This statement should provide the following information, where applicable:

- Accession codes, unique identifiers, or web links for publicly available datasets
- A description of any restrictions on data availability
- For clinical datasets or third party data, please ensure that the statement adheres to our policy

The single-cell RNA-seq and bulk RNA-seq data has been deposited at NCBI GEO under the accession code GSE182176 (https://www.ncbi.nlm.nih.gov/query/

acc.cgi?acc=GSE182176). The flow cytometry-based data generated in this study are provided in the Source Data file associated with this manuscript. The Microscopy data reported in this paper will be shared by the lead contact upon request. This paper does not report original code. Any additional information required to re-analyze the data reported in this paper is available from the lead contact upon request.
The single-cell RNA-seq and bulk RNA-seq data has been deposited at NCBI GEO under the accession code GSE182176 (https://www.ncbi.nlm.nih.gov/query/acc.cgi?acc=GSE182176). The flow cytometry-based data generated in this study are provided in the Source Data file associated with this manuscript. The Microscopy data reported in this paper will be shared by the lead contact upon request. This paper does not report original code. Any additional information required to re-analyze the data reported in this paper is available from the lead contact upon request.
Data and Code availability
The single-cell RNA-seq and bulk RNA-seq data has been deposited at NCBI GEO under the accession code GSE182176 (https://www.ncbi.nlm.nih.gov/query/acc.cgi?acc=GSE182176). The flow cytometry-based data generated in this study are provided in the Source Data file associated with this manuscript. The Microscopy data reported in this paper will be shared by the lead contact upon request. This paper does not report original code. Any additional information required to re-analyze the data reported in this paper is available from the lead contact upon request.

Human research participants

Policy information about studies involving human research participants and Sex and Gender in Research.

Reporting on sex and gender	Not applicable
Population characteristics	*Describe the covariate-relevant population characteristics of the human research participants (e.g. age, genotypic information, past and current diagnosis and treatment categories). If you filled out the behavioural & social sciences study design questions and have nothing to add here, write "See above."*
Recruitment	*Describe how participants were recruited. Outline any potential self-selection bias or other biases that may be present and how these are likely to impact results.*
Ethics oversight	*Identify the organization(s) that approved the study protocol.*

Note that full information on the approval of the study protocol must also be provided in the manuscript.

Field-specific reporting

Please select the one below that is the best fit for your research. If you are not sure, read the appropriate sections before making your selection.

☒ Life sciences ☐ Behavioural & social sciences ☐ Ecological, evolutionary & environmental sciences

For a reference copy of the document with all sections, see nature.com/documents/nr-reporting-summary-flat.pdf

Life sciences study design

All studies must disclose on these points even when the disclosure is negative.

Sample size	We did not perform specific power calculations, but wherever possible had a minimum of 3 mice/group and experiment and all experiments were repeated 2-3 times. This was restricted by the number of animals available with the correct genotype. In the experiments involving 4-OHT injection into pregnant mice, we not know the genotype of the embryos before analysis and thus some groups in some experiments contained less than 3 mice. These sets of experiments were however repeated 4 times. For scRNA-seq of colon transplants 3 colonic grafts were pooled prior to analysis. This was deemed sufficient as the purpose of these experiments was to determine whether embryonic fibroblast could give rise to fibroblast subsets that were transcriptionally and phenotypically related to those found the intestine of adult mice.
Data exclusions	In the experiments depicted in Figure S4A, the cells from one large intestinal sample died during preparation and were not included in the analysis. In Figure S4E we excluded one Cre- EPCAM+ small intestinal sample outlier, where for some unknown reason 24% of cells were YFP+. YFP positive cells among EPCAM+ cells in 9 other Cre- mouse embryos was less that 0.3%.
Replication	All flow cytometry and scRNA-seq experiments were repeated at least 2-3 times except the scRNA-seq of colon transplants as described above. All attempts at replication were successful.
Randomization	Mice were grouped based on genotype.
Blinding	For Flow cytometry data collection the experimentalist was most often aware of the source of the data being analysed. Blinding was not deemed necessary as data derived through the flow cytometer is not subjective.<br>Blinding was not either deemed necessary for the bioinformatics analysis of scRNA-seq data.

Reporting for specific materials, systems and methods

We require information from authors about some types of materials, experimental systems and methods used in many studies. Here, indicate whether each material, system or method listed is relevant to your study. If you are not sure if a list item applies to your research, read the appropriate section before selecting a response.

Materials & experimental systems

n/a	Involved in the study
☐	☒ Antibodies
☒	☐ Eukaryotic cell lines
☒	☐ Palaeontology and archaeology
☐	☒ Animals and other organisms
☒	☐ Clinical data
☒	☐ Dual use research of concern

Methods

n/a	Involved in the study
☒	☐ ChIP-seq
☐	☒ Flow cytometry
☒	☐ MRI-based neuroimaging

Antibodies

Antibodies used

AF488 donkey anti-rat IgG Jackson IR Cat#712-545-153, RRID: AB_2340684
AF488 anti-mouse αSMA clone 1A4 Abcam Cat#ab184675, RRID: AB_2832195
AF488 streptavidin Thermo Fischer Scientific (TFS) Cat#S32354, RRID: AB_2315383
AF594 anti-mouse CD31 clone MEC13.3 BioLegend Cat#102520, RRID: AB_2563319
AF647 anti-mouse CD31 clone MEC13.3 BioLegend Cat#102515, RRID: AB_2161030
AF647 donkey anti-goat IgG Jackson IR Cat#705-605-147, RRID: AB_2340437
AF647 donkey anti-rabbit IgG Jackson IR Cat#711-605-152, RRID: AB_2492288
AF647 anti-mouse EpCAM clone G8.8 BioLegend Cat#118211, RRID: AB_1134104
AF700 anti-mouse B220 clone RA3-6B2 BioLegend Cat#103232; RRID: AB_493717
AF700 anti-mouse CD11b clone M1/70 BioLegend Cat#101222, RRID: AB_493705
AF700 anti-mouse CD11c clone N418 BioLegend Cat#117320, RRID: AB_528736
AF700 anti-mouse CD45 clone 30-F11 TFS Cat#56-0451-82, RRID: AB_891454
AF700 anti-mouse CD45.2 clone 104 TFS Cat#56-0454-82, RRID: AB_657752
AF700 anti-mouse Gr-1 clone RB6-8C5 BioLegend Cat#108422, RRID: AB_2137487
AF700 anti-mouse NK1.1 clone PK136 BioLegend Cat#108730, RRID: AB_2291262
AF700 anti-mouse Ter119 clone Ter119 BioLegend Cat#116220, RRID: AB_528963
APC anti-mouse BP3 clone BP3 BioLegend Cat#140208, RRID: AB_10901172
APC anti-mouse L1CAM clone 555 Miltenyi Cat#130-102-221, RRID: AB_2655594
APC anti-mouse NCAM clone 809220 R&D Cat#FAB7820A
APCCy7 anti-mouse CD90.2 clone 53-2.1 BD Biosciences Cat#561641, RRID: AB_10898013
APCCy7 anti-mouse CD45.2 clone 104 BioLegend Cat#109824, RRID: AB_830789
APCCy7 anti-mouse EpCAM clone G8.8 BioLegend Cat#118218, RRID: AB_2098648
APC-eF780 anti-mouse Ter119 clone Ter119 TFS Cat#47-5921-82, RRID: AB_1548786
Biotin anti-mouse CD34 clone MEC14.7 BioLegend Cat#119304; RRID: AB_345282
Biotin anti-mouse CD81 clone Eat-2 BioLegend Cat#104903, RRID: AB_313138
BUV395 anti-mouse CD146 clone ME-9F1 BD Biosciences Cat#740330, RRID: AB_2740063
BV421 anti-mouse PDGFRα clone APA5 BD Biosciences Cat#566293, RRID: AB_2739666
BV421 anti-mouse CD34 clone RAM34 BD Biosciences Cat#562608, RRID: AB_11154576
BV510 anti-mouse EpCAM clone G8.8 BD Biosciences Cat#747748, RRID: AB_2872217
BV510 streptavidin BD Biosciences Cat#563261, RRID: AB_2869477
BV605 anti-mouse CD31 clone 390 BioLegend Cat#102427, RRID: AB_2563982
BV605 anti-mouse Itgb1 clone HM β1-1 BD Biosciences Cat#740365, RRID: AB_2740097
BV650 anti-mouse BP3 clone BP-3 BD Biosciences Cat#740611, RRID: AB_2740311
BV650 anti-mouse CD31 clone 390 BD Biosciences Cat#740483, RRID: AB_2740207
BV711 anti-mouse CD9 clone KMC8 BD Biosciences Cat#740696, RRID: AB_2740380
BV786 anti-mouse BP3 clone BP-3 BD Biosciences Cat#741012, RRID: AB_2740634
Cy3 donkey anti-rat IgG Jackson IR Cat#712-166-150, RRID: AB_2340668
Cy3 goat anti Syrian hamster IgG Jackson IR Cat#107-165-142, RRID: AB_2337464
FITC anti-mouse CD34 clone RAM34 TFS Cat#11-0341-85, RRID: AB_465022
FITC anti-mouse CD90.2 clone 53-2.1 TFS Cat#11-0902-82, RRID: AB_465154
PE anti-mouse CD141 clone REA964 Miltenyi Cat#130-116-017, RRID: AB_2727308
PE anti-mouse CD26 clone H194-112 BioLegend Cat#137804, RRID: AB_2293047
PE anti-mouse ESAM clone 1G8 BioLegend Cat#136204, RRID: AB_1953301
PE anti-mouse Itgb1 clone HMb1-1 BioLegend Cat#102208, RRID: AB_312885
PE/CF594 anti-mouse PDGFRα clone APA5 BD Biosciences Cat#562775, RRID: AB_2737786
PECy7 anti-mouse PDPN clone 8.1.1 TFS Cat#25-5381-82, RRID: AB_2573460
PerCP/Cy5.5 anti-mouse CD31 clone 390 BioLegend Cat#102420, RRID: AB_10613644
PerCP-eF710 anti-mouse EpCAM clone G8.8 TFS Cat#46-5791-82, RRID: AB_10598205
Unconjugated anti-mouse CD34 clone RAM34 TFS Cat#14-0341-82, RRID: AB_467210
Unconjugated anti mouse PDPN clone 8.1.1 Biolegend Cat#127402, RRID: AB_1089187
Unconjugated anti-mouse PPARγ polyclonal Invitrogen Cat#PA5-25757, RRID: AB_2543257
Unconjugated anti-mouse PDGFRα polyclonal R&D Cat#AF1062, RRID: AB_2236897

Validation

All antibodies came from commercial vendors. Validatio nof individual antibodies can be found in the links below.

AF488 donkey anti-rat IgG https://www.jacksonimmuno.com/catalog/products/712-545-153
AF488 anti-mouse αSMA clone 1A4 https://www.abcam.com/products/primary-antibodies/alexa-fluor-488-alpha-smooth-muscle-actin-antibody-1a4-ab184675.html
AF488 streptavidin https://www.thermofisher.com/order/catalog/product/S32354?SID=srch-hj-S32354
AF594 anti-mouse CD31 clone MEC13.3 https://www.biolegend.com/en-us/products/alexa-fluor-594-anti-mouse-cd31-antibody-9633
AF647 anti-mouse CD31 clone MEC13.3 https://www.biolegend.com/en-us/products/alexa-fluor-647-anti-mouse-cd31-antibody-3094
AF647 donkey anti-goat IgG https://www.jacksonimmuno.com/catalog/products/705-605-147
AF647 donkey anti-rabbit IgG https://www.jacksonimmuno.com/catalog/products/711-605-152
AF647 anti-mouse EpCAM clone G8.8 https://www.biolegend.com/en-us/products/alexa-fluor-647-anti-mouse-cd326-ep-cam-antibody-4973
AF700 anti-mouse B220 clone RA3-6B2 https://www.biolegend.com/en-us/products/alexa-fluor-700-anti-mouse-human-cd45r-b220-antibody-3408
AF700 anti-mouse CD11b clone M1/70 https://www.biolegend.com/en-us/products/alexa-fluor-700-anti-mouse-human-cd11b-antibody-3388
AF700 anti-mouse CD11c clone N418 https://www.biolegend.com/en-us/products/alexa-fluor-700-anti-mouse-cd11c-antibody-3429
AF700 anti-mouse CD45 clone 30-F11 https://www.thermofisher.com/antibody/product/CD45-Antibody-clone-30-F11-Monoclonal/56-0451-82
AF700 anti-mouse CD45.2 clone 104 https://www.thermofisher.com/antibody/product/CD45-2-Antibody-clone-104-Monoclonal/56-0454-82
AF700 anti-mouse Gr-1 clone RB6-8C5 https://www.biolegend.com/en-us/products/alexa-fluor-700-anti-mouse-ly-6g-ly-6c-gr-1-antibody-3390
AF700 anti-mouse NK1.1 clone PK136 https://www.biolegend.com/en-us/products/alexa-fluor-700-anti-mouse-nk-1-1-antibody-6555
AF700 anti-mouse Ter119 clone Ter119 https://www.biolegend.com/en-us/products/alexa-fluor-700-anti-mouse-ter-119-erythroid-cells-antibody-3428.
APC anti-mouse BP3 clone BP3 https://www.biolegend.com/en-us/products/apc-anti-mouse-cd157-bst-1-antibody-7272
APC anti-mouse L1CAM clone 555 https://www.miltenyibiotec.com/DK-en/products/cd171-l1cam-antibody-anti-mouse-555.html#conjugate=apc:size=30-ug-in-1-ml.
APC anti-mouse NCAM clone 809220 https://www.rndsystems.com/products/mouse-ncam-1-cd56-apc-conjugated-antibody-809220_fab7820a
APCCy7 anti-mouse CD90.2 clone 53-2.1 https://www.bdbiosciences.com/en-us/products/reagents/flow-cytometry-reagents/research-reagents/single-color-antibodies-ruo/apc-cy-7-rat-anti-mouse-cd90-2.561641
APCCy7 anti-mouse CD45.2 clone 104 https://www.biolegend.com/en-us/products/apc-cyanine7-anti-mouse-cd45-2-antibody-3906
APCCy7 anti-mouse EpCAM clone G8.8 https://www.biolegend.com/en-us/products/apc-cyanine7-anti-mouse-cd326-ep-cam-antibody-5577
APC-eF780 anti-mouse Ter119 clone Ter119 https://www.thermofisher.com/antibody/product/TER-119-Antibody-clone-TER-119-Monoclonal/47-5921-82
Biotin anti-mouse CD34 clone MEC14.7 https://www.biolegend.com/en-us/products/biotin-anti-mouse-cd34-antibody-2602
Biotin anti-mouse CD81 clone Eat-2 https://www.biolegend.com/en-us/products/biotin-anti-mouse-rat-cd81-antibody-236
BUV395 anti-mouse CD146 clone ME-9F1 https://www.bdbiosciences.com/en-us/products/reagents/flow-cytometry-reagents/research-reagents/single-color-antibodies-ruo/buv395-rat-anti-mouse-cd146.740330
BV421 anti-mouse PDGFRα clone APA5 https://www.bdbiosciences.com/en-us/products/reagents/flow-cytometry-reagents/research-reagents/single-color-antibodies-ruo/bv421-rat-anti-mouse-cd140a.566293
BV421 anti-mouse CD34 clone RAM34 https://www.bdbiosciences.com/en-us/products/reagents/flow-cytometry-reagents/research-reagents/single-color-antibodies-ruo/bv421-rat-anti-mouse-cd34.562608
BV510 anti-mouse EpCAM clone G8.8 BD Biosciences Cat#747748, RRID: AB_2872217 https://www.bdbiosciences.com/en-us/products/reagents/flow-cytometry-reagents/research-reagents/single-color-antibodies-ruo/bv510-rat-anti-mouse-cd326.747748
BV510 streptavidin https://www.bdbiosciences.com/en-us/products/reagents/flow-cytometry-reagents/research-reagents/single-color-antibodies-ruo/bv510-streptavidin.563261
BV605 anti-mouse CD31 clone 390 https://www.biolegend.com/en-us/products/brilliant-violet-605-anti-mouse-cd31-antibody-9963
BV605 anti-mouse Itgb1 clone HM β1-1 https://www.bdbiosciences.com/en-us/products/reagents/flow-cytometry-reagents/research-reagents/single-color-antibodies-ruo/bv605-hamster-anti-mouse-cd29.740365
BV650 anti-mouse BP3 clone BP-3 https://www.bdbiosciences.com/en-us/products/reagents/flow-cytometry-reagents/research-reagents/single-color-antibodies-ruo/bv650-mouse-anti-mouse-cd157.740611
BV650 anti-mouse CD31 clone 390 https://www.bdbiosciences.com/en-us/products/reagents/flow-cytometry-reagents/research-reagents/single-color-antibodies-ruo/bv650-rat-anti-mouse-cd31.740483
BV711 anti-mouse CD9 clone KMC8 https://www.bdbiosciences.com/en-us/products/reagents/flow-cytometry-reagents/research-reagents/single-color-antibodies-ruo/bv711-rat-anti-mouse-cd9.740696
BV786 anti-mouse BP3 clone BP-3 https://www.bdbiosciences.com/en-us/products/reagents/flow-cytometry-reagents/research-reagents/single-color-antibodies-ruo/bv786-mouse-anti-mouse-cd157.741012
Cy3 donkey anti-rat IgG https://www.jacksonimmuno.com/catalog/products/712-166-150
Cy3 goat anti Syrian hamster IgG https://www.jacksonimmuno.com/catalog/products/107-165-142
FITC anti-mouse CD34 clone RAM34 https://www.thermofisher.com/antibody/product/CD34-Antibody-clone-RAM34-Monoclonal/11-0341-85
FITC anti-mouse CD90.2 clone 53-2.1 https://www.thermofisher.com/antibody/product/CD90-2-Thy-1-2-Antibody-clone-53-2-1-Monoclonal/11-0902-82
PE anti-mouse CD141 clone REA964 https://www.miltenyibiotec.com/DK-en/products/cd141-bdca-3-antibody-anti-mouse-reafinity-rea964.html#conjugate=biotin:size=150-ug-in-1-ml
PE anti-mouse CD26 clone H194-112 https://www.biolegend.com/en-us/products/pe-anti-mouse-cd26-dpp-4-antibody-6551
PE anti-mouse ESAM clone 1G8 https://www.biolegend.com/en-us/products/pe-anti-mouse-esam-antibody-6287
PE anti-mouse Itgb1 clone HMb1-1 https://www.biolegend.com/en-us/products/pe-anti-mouse-rat-cd29-antibody-2259
PE/CF594 anti-mouse PDGFRα clone APA5 https://www.bdbiosciences.com/en-us/products/reagents/flow-cytometry-reagents/research-reagents/single-color-antibodies-ruo/pe-cf594-rat-anti-mouse-cd140a.562775
PECy7 anti-mouse PDPN clone 8.1.1 https://www.thermofisher.com/antibody/product/Podoplanin-Antibody-clone-eBio8-1-1-8-1-1-Monoclonal/25-5381-82

PerCP/Cy5.5 anti-mouse CD31 clone 390 https://www.biolegend.com/en-us/products/percp-cyanine5-5-anti-mouse-cd31-antibody-6668
PerCP-eF710 anti-mouse EpCAM clone G8.8 https://www.thermofisher.com/antibody/product/CD326-EpCAM-Antibody-clone-G8-8-Monoclonal/46-5791-82
Unconjugated anti-mouse CD34 clone RAM34 https://www.thermofisher.com/antibody/product/CD34-Antibody-clone-RAM34-Monoclonal/14-0341-82
Unconjugated anti mouse PDPN clone 8.1.1 https://www.biolegend.com/en-us/products/purified-anti-mouse-podoplanin-antibody-4749
Unconjugated anti-mouse PPARγ polyclonal https://www.thermofisher.com/antibody/product/PPAR-gamma-Antibody-Polyclonal/PA5-25757
Unconjugated anti-mouse PDGFRα polyclonal https://www.rndsystems.com/products/mouse-pdgf-ralpha-antibody_af1062

Animals and other research organisms

Policy information about studies involving animals; ARRIVE guidelines recommended for reporting animal research, and Sex and Gender in Research

Laboratory animals

Littermates were used for all experiments. Both males and females were used in experiments and evenly distributed amongst the groups where possible. For flow cytometry analysis adult mice were used between 5.5-12w of age. For scRNA-seq analysis of adult intestine females aged 8-10 weeks were used. Pregnant mothers were injected with 4-OH at E11.5. Flow cytometry of mouse embryos was performed at E12.5 and E13.5.

Mouse strains:
Gli1-CreERT2: Gli1tm3(cre/ERT2)Alj The Jackson Laboratory, IMSR_JAX:007913
R26R.EYFP: B6.129X1-Gt(ROSA)26Sortm1(EYFP)Cos/J The Jackson Laboratory, IMSR_JAX:006148
Gli1-EGFP (Garcia et al., 2010)
EYFP: (obtained by crossing R26R.EYFP with the relevant Cre mice).
Ackr4.EGFP: Ackr4tmlCcbl1 (Heinzel, Benz and Bleul, 2007), MGI: 5316751
C57BL/6Nrj mice were purchased from Janvier Labs (Le Genest-Saint-Isle, France)

Wild animals

Wild animals were not part of this study.

Reporting on sex

For the labeling and lineage tracing experiments we did not split groups based on sex because of limited numbers of mice. Male and female mice were evenly distributed between groups where possible. Female mice alone were used for scRNA seq analysis of adult FB, as our purpose was to examine differences between small intestine and colon MSC subsets and not differences between sex.

Field-collected samples

Samples were not collected in the field.

Ethics oversight

All experiments in Denmark were approved by the Danish Animal Experiments Inspectorate, and experiments conducted in Glasgow were performed with ethical approval under a Project Licence from the the UK Home Office.

Note that full information on the approval of the study protocol must also be provided in the manuscript.

Flow Cytometry

Plots

Confirm that:

☒ The axis labels state the marker and fluorochrome used (e.g. CD4-FITC).

☒ The axis scales are clearly visible. Include numbers along axes only for bottom left plot of group (a 'group' is an analysis of identical markers).

☒ All plots are contour plots with outliers or pseudocolor plots.

☒ A numerical value for number of cells or percentage (with statistics) is provided.

Methodology

Sample preparation

Small intestinal and large intestinal tissue samples were obtained from adult, embryo or transplanted intestines. Washed intestinal tissue was opened longitudinally and Peyer's patches removed. For scRNA-seq and bulk RNA-seq experiments on adult intestine, muscularis externa was stripped away using tweezers. Tissues were cut into 0.5-1 cm pieces and epithelial cells removed by 3 consecutive rounds of incubation in HBSS supplemented with HEPES, sodium pyruvate, penicillin/streptomycin, gentamycin, EDTA and FCS, for 15 min at 37 degrees C with constant shaking at 350 rpm. After each incubation, samples were shaken for 10 sec and medium containing epithelial cells and debris was discarded. For colonic tissues, DL-dithiothreitol was added at the first incubation step. Remaining tissue pieces were digested with collagenase P or with Liberase TM and DNAse I in R10 medium for up to 30 min at 37 degrees C with constant shaking at 550 rpm (small intestine) or with a magnetic stirrer and at 280 rpm (large intestine). For bulk RNA-seq cells were treated with ACK lysing buffer to lyse red blood cells prior to sorting. For isolation of cells from embryonic intestine, tissues were digested directly for 30 min at 37 degrees C in Eppendorf tubes with constant shaking at 900 rpm. The resulting cell suspensions were filtered through a 70 µm filter and washed in MACS buffer twice prior to subsequent analyses.

For flow cytometry:
Cell suspensions were stained with fluorochrome labelled primary antibodies in Brilliant stain buffer for 30 min on ice before analyzed by Flow Cytometry. Dead cells were identified by staining with either 7-AAD or Zombie UV fixable viability dye and cell doublets were excluded on the basis of FSC-A/FSC-H. For intracellular staining, cells were stained for surface antigens, fixed with FoxP3 Staining Buffer set and stained for αSMA in FoxP3 Permeabilization buffer. After washing, cells were stained with antibodies to surface antigens not compatible with fixation according to the manufacturer's instructions.

Instrument	Data was acquired on an LSR Fortessa II, FACSAria Fusion or FACSMelody (BD Biosciences).
Software	Data was analyzed using the FlowJo software (version 10.6)
Cell population abundance	For flow cytometry cell sorting and subsequent scRNA-seq analysis post sort purity ranged from 58-85%. All contaminating cell types were removed computationally prior to further analysis.
Gating strategy	Cell debris and cell aggregates were excluded by FSC-A/SSC-A and FSC-A/FSC-H gating. Dead cells were identified by positive staining with 7-AAD or Zombie UV fixable viability dye and excluded from analysis. Gates were set manually and examples of flow cytometry analysis of adult, embryonic and kidney transplant intestine can be found in the supplementary data. When analysing YFP+ or GFP+ mice YFP- or GFP- mice were used as negative controls.

☒ Tick this box to confirm that a figure exemplifying the gating strategy is provided in the Supplementary Information.
